# Research co-design in health: a rapid overview of reviews

**DOI:** 10.1186/s12961-020-0528-9

**Published:** 2020-02-11

**Authors:** Peter Slattery, Alexander K. Saeri, Peter Bragge

**Affiliations:** BehaviourWorks Australia, Monash Sustainable Development Institute, Monash University, Melbourne, Australia

**Keywords:** Research co-design, patient and public involvement, research engagement, community–academic partnership, participatory research

## Abstract

**Background:**

Billions of dollars are lost annually in health research that fails to create meaningful benefits for patients. Engaging in research co-design – the meaningful involvement of end-users in research – may help address this research waste. This rapid overview of reviews addressed three related questions, namely (1) what approaches to research co-design exist in health settings? (2) What activities do these research co-design approaches involve? (3) What do we know about the effectiveness of existing research co-design approaches? The review focused on the study planning phase of research, defined as the point up to which the research question and study design are finalised.

**Methods:**

Reviews of research co-design were systematically identified using a rapid overview of reviews approach (PROSPERO: CRD42019123034). The search strategy encompassed three academic databases, three grey literature databases, and a hand-search of the journal *Research Involvement and Engagement*. Two reviewers independently conducted the screening and data extraction and resolved disagreements through discussion. Disputes were resolved through discussion with a senior author (PB). One reviewer performed quality assessment. The results were narratively synthesised.

**Results:**

A total of 26 records (reporting on 23 reviews) met the inclusion criteria. Reviews varied widely in their application of ‘research co-design’ and their application contexts, scope and theoretical foci. The research co-design approaches identified involved interactions with end-users outside of study planning, such as recruitment and dissemination. Activities involved in research co-design included focus groups, interviews and surveys. The effectiveness of research co-design has rarely been evaluated empirically or experimentally; however, qualitative exploration has described the positive and negative outcomes associated with co-design. The research provided many recommendations for conducting research co-design, including training participating end-users in research skills, having regular communication between researchers and end-users, setting clear end-user expectations, and assigning set roles to all parties involved in co-design.

**Conclusions:**

Research co-design appears to be widely used but seldom described or evaluated in detail. Though it has rarely been tested empirically or experimentally, existing research suggests that it can benefit researchers, practitioners, research processes and research outcomes. Realising the potential of research co-design may require the development of clearer and more consistent terminology, better reporting of the activities involved and better evaluation.

## Background

The ultimate aim of health research is to provide evidence and insights that can be used to improve health outcomes. However, Glasziou and Chalmers [1, 2] estimate that 85% of funding for medical research – a staggering $170 billion annually – is avoidably wasted through non-publication, incomplete reporting and poor design.

A key contributor to this waste, aside from publication and reporting issues, is that health research frequently addresses questions and outcomes of limited relevance to clinicians, patients and other end-users [3]. For example, Oliver and Gray [4] found that only 9 of 334 studies compared researchers’ priorities with those of patients or practitioners. This means that a significant proportion of health research is potentially wasted from the outset, because researchers have not consulted with patients, clinicians and other end-users when prioritising an area of research or selecting a specific research question [1]. The resulting gap between research and end-user needs is underscored by Ioannidis, who points out that “*practicing doctors and other health care professionals* [are] *familiar with how little of what they find in medical journals is useful*” ([3], p. 1), and suggests that a lack of pragmatism and a lack of patient centeredness are two of the major reasons for research ‘waste’.

In response to this need, organisations and initiatives such as INVOLVE in the United Kingdom, the James Lind Alliance (United Kingdom, established in 2004) and the Patient Centred Outcomes Research Institute (PCORI) (United States, established in 2010) promote the involvement of clinicians, patients and other health service end-users in the health research process (e.g. [5–7]).

In parallel with the establishment of patient-centred research organisations, researchers have themselves examined different methods of health stakeholder engagement (e.g. [8–10]). Methods for stakeholder engagement and collaborative data collection have been developed and applied across a range of populations, including the elderly and intellectually disabled (e.g. [11–13]). Numerous collaborative health research projects have been conducted and evaluated, ranging from projects involving limited engagement by co-design participants to those led by end-users, for example, where activists engaged researchers [10]. A wide range of practices for how to manage data collection (e.g. interviews, participation in advisory councils) and researcher and co-design participant communication and relationships have also been recommended (e.g. [5, 14, 15]). However, the current literature is complex, contradictory and poorly synthesised as it has been examined from several different research perspectives in studies focused on maximising rigor and completeness for academic understanding rather than on providing practitioners with a short, parsimonious and accessible synthesis of the most significant characteristics of the literature.

The aim of this study was to provide an accessible synopsis of current co-design approaches and activities in health research by reviewing all reviews of relevant literature. Its key audience is health funders, policy-makers and practitioners who need to know which co-design approaches and activities to include in research programmes. In particular, we focus on research pertaining to patient, clinician and other end-user engagement in health research during the research planning phase, where the research topics and agendas are set, research questions and aims are agreed, and study design and materials are finalised. Our rationale for focusing on the study planning phase is that involvement of end-user groups in setting the research question (and/or the wider research agenda) is particularly critical for avoiding research waste [3].

The specific review questions were:

1. What approaches to research co-design exist in health settings?
2. What activities do these research co-design approaches involve?
3. What do we know about the effectiveness of existing research co-design approaches?

This review was undertaken as part of a larger project to develop and test a health research co-design process that maximises alignment between researchers, clinicians and patients when developing a research question. Project funding was provided by the Victorian Transport Accident Commission, Australia.

## Method

A ‘rapid overview of reviews’ approach was used. Rapid overviews of reviews are a type of rapid review, an emerging approach to research synthesis that utilises systematic search and appraisal processes but, unlike systematic reviews, focuses on review-level rather than primary studies [16, 17]. Rapid overviews of reviews aim to synthesise the questions addressed by systematic reviews and capture relevant insights [17]. The review was pre-registered with the international prospective register of systematic reviews (PROSPERO: CRD42019123034). Deviations from this protocol are listed in Additional file 1. The Preferred Reporting Items for Systematic Reviews and Meta-Analyses (PRISMA) Statement has been used as a reporting framework [18].

### Definitions

Co-design is meaningful end-user engagement in research design and includes instances of engagement that occur across all stages of the research process and range in intensity from relatively passive to highly active and involved. The definitions used to scope this review were informed by the PCORI, a research funding organisation that has developed a taxonomy of research process phases, stakeholder groups and other concepts relevant to the review. We therefore defined ‘research co-design’ as the meaningful involvement of research users during the study planning phase of a research project, where ‘meaningful involvement’ is taken to refer to participation in an explicitly described, defined and auditable role or task necessary to the planning and/or conduct of health research. We defined ‘research users’ as consumers, clinicians or other people or groups (other than researchers themselves) that have an interest in the results of health research. We defined the ‘study planning phase’ as all activities occurring prior to the finalisation of the research question in a research study. Based on our definition, studies that do not encompass the research planning phase, for example, research user involvement in participant recruitment into a research project, research activities such as data collection and analysis, and dissemination and translation of research findings, are excluded from this review. The rationale for focusing on the study planning phase is that reaching a shared understanding of the research question (that is, the problem to be addressed) is a critical point in the research process as decisions made at this stage will influence all subsequent research processes. The study planning phase is therefore the point where research waste has the most potential to be averted.

### Search process

The following academic databases were searched: MEDLINE (1946 to 10 January 2019), PsycINFO (1806 to 10 January 2019), Cochrane Database of Systematic Reviews (inception to 10 January 2019). The following grey literature databases were hand searched: PCORI (24 January 2019), INVOLVE (24 January 2019), Health Systems Evidence (24 January 2019) and James Lind Alliance (24 January 2019). We directly searched the journal *Research Involvement and Engagement* (2014 to 10 January 2019) as its scope is directly relevant to the review questions and it was not indexed in MEDLINE at the time of the search.

### Screening and selection of studies

Our inclusion criteria were:

1. Systematic or narrative reviews (quantitative or qualitative studies) of research co-design (as defined above). Reviews had to address at least one of the following (adapted from PCORI classifications [19]):
  - Examples of research co-design (e.g. review of primary studies where engagement took place); and/or
  - Description of research co-design methodologies (e.g. synthesis and presentation of framework for research engagement); and/or
  - Evaluation of research co-design (e.g. a meta-analysis of engagement effectiveness in influencing patient outcomes or experiences)
2. English language
3. Peer-reviewed journal publications or publicly available reports

Our exclusion criteria were:

1. Primary studies
2. Non-health settings
3. Reviews describing research user engagement:
  - in non-research processes or projects (e.g. engagement in healthcare)
  - only outside the study planning phase (i.e. after the point at which the research question has been finalised)
4. Reviews describing engagement with non-research stakeholders where there is no identified interest in a specific research project (e.g. public submissions on research priorities)

We searched using a combination of defined search terms and subject headings (where available). Examples of our search strings are included in Additional file 2.

Two reviewers (PS, AS) conducted abstract screening independently. Disputes were resolved through discussion between reviewers. Two reviewers (PS, AS) conducted full text screening independently. Disputes were resolved first through discussion between reviewers, with a senior author (PB) resolving disagreements.

### Quality appraisal

One reviewer (PS) performed quality assessment on all systematic reviews using the Assessing the Methodological Quality of Systematic Reviews (AMSTAR) checklist [20]. This is a well-established tool for assessing the quality of systematic reviews and meta-analyses [21]. As AMSTAR is only designed for the evaluation of systematic reviews, quality assessment on the non-systematic reviews was not conducted.

### Data extraction

Two reviewers (PS & AS) conducted data extraction independently. This focused on extracting (1) study information, (2) content relevant to our three research questions, and (3) recommendations. One reviewer (PS) then evaluated and synthesised both sets of data. This reviewer resolved disagreements by examining the original article. The data extracted is available in Additional files 3 and 5.

### Analysis

The nature of the research questions and the heterogeneity of the review results precluded a quantitative analysis. The output of the review is therefore a narrative summary of the results, focused on their contribution to each research question. This summary was developed through the evaluation of both reviewers (PS & AS) who conducted screening and data extraction. The presentation of results distinguishes, where appropriate, between reviews with a high AMSTAR rating (5–7), those with a low AMSTAR rating (3–4), and non-systematic reviews.

## Results

**Fig. 1:**
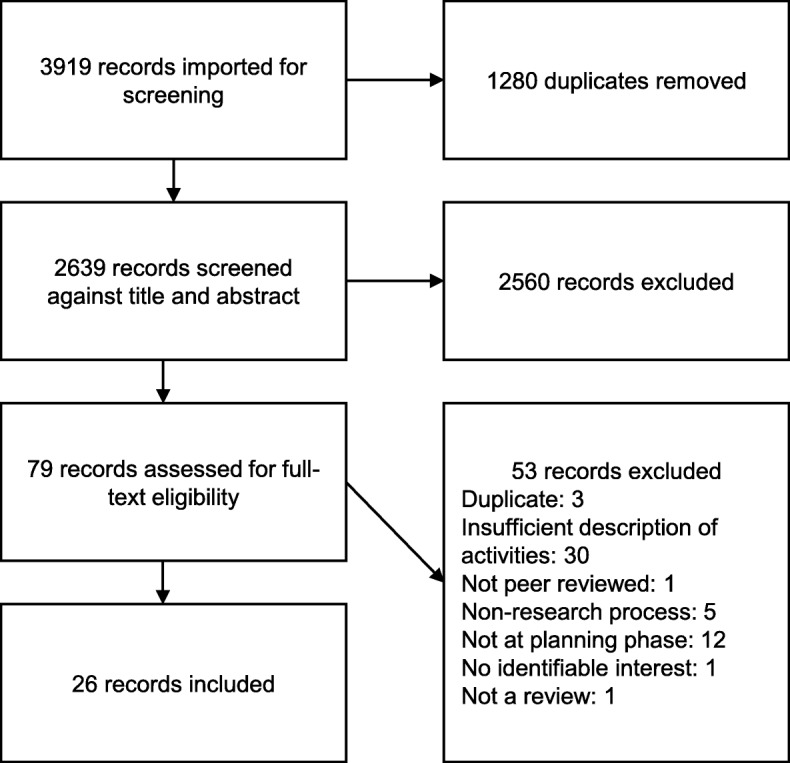
PRISMA flow diagram for rapid overview of reviews in health research co-design

A total of 3919 records were identified across all searches; 1280 records were duplicates and removed automatically using Covidence, an online tool that assists the conduct of systematic reviews [22]. The abstracts of the remaining 2639 records were independently screened by two reviewers (PS and AS), with 2560 records being excluded at this stage and 53 records being excluded at the full text stage. Disagreements were resolved through discussion. The remaining 26 records were finally included. Figure 1 illustrates this process using a PRISMA flow diagram.

The final 26 records included 23 reviews (3 pairs of records reported on the same review). These comprised 8 systematic reviews, 2 scoping reviews, 4 narrative reviews and 9 other review types, including systematic literature scans and critical reviews. Two were unpublished reports. Additional file 3 describes these studies in greater detail; Additional file 4 describes records excluded at full text review and reasons for exclusion; and Additional file 5 provides the extraction tables.

### Quality appraisal

Thirteen studies were systematic reviews and were therefore appraised using the AMSTAR tool. The results of quality appraisal with AMSTAR for these 13 studies are shown in Additional file 6. Of the total possible score of 11/11 for AMSTAR criteria satisfied, the studies ranged from 3 to 7 in score, with a mean of 5. Only 3 of the reviewed studies provided an a priori design and only 5 included grey literature. Our findings should be interpreted with due consideration of the quality of the reviews included.

### What approaches to research co-design exist in health settings?

Our review described many approaches to research co-design that encompassed our definition of research co-design. There were no distinct differences between high-quality (AMSTAR ≥5), lower-quality, and non-systematic reviews in the research co-design approaches discussed. Author definitions of these research co-design approaches are presented in Table 1.

**Table 2:** For better research, INVVOLVE research end-users in co-design

*I*nvest in co-design	Allocate sufficient time and resources Pay/reward participants for their time Provide training, if needed
*N*eeds assessment	Determine project co-design needs: why, how and on what will co-design participants and researchers collaborate?
*V*ision roles, responsibilities and rewards	Set clear roles and responsibilities of all participants in co-design Clarify how co-design participant feedback will be used Ensure all parties understand the importance of co-design and the potential benefits Manage expectations – make sure that there is a shared vision and goal
*V*alidate participants	Empower and nurture participants so that they are confident enough to engage with researchers and the research process
*O*rganise interaction carefully	Make sure meeting places are accessible and familiar Make sure that any interactions are well structured and regular Regularly communicate and update all parties Prepare aids, such as glossaries, images and plans, as meeting facilitators Have backup co-design participants as some may drop out
*L*ead the engagement	Carefully define and control the scope of engagement Don’t let groups dominate conversations and decision-making Discuss and defuse tensions
*V*alue patient time and input	Build trust and rapport between researchers and co-design participants Give co-design participants some choice and control
*E*valuate and report	Document all engagement processes Evaluate processes and outcomes based on predetermined criteria Report findings

The research co-design approaches described in the literature (Table 1) overlapped significantly in scope. All focused on some type of research-related engagement with one or many groups of research end-users, such as the ‘public’ [23, 30], ‘patient’ [5], ‘consumer’ [10, 26], ‘stakeholder’ [25] and ‘communities’ [15]. Most used the terms ‘involvement’ [8, 12, 31, 32] or ‘engagement’ [5] to describe cases where research end-users contributed to the research process.

However, there were also many differences across these approaches. Some approaches were specifically focused on older adults [12], children [31] or adults with intellectual disabilities [32]. For instance, inclusive health research is limited to “*research which includes or involves people with learning disabilities as more than just subjects of research*” ([27], p. 275), while patient and public involvement is described much more broadly as “*doing research ‘with’ or ‘by’ the public, rather than ‘to’, ‘about’ or ‘for’ the public*” ([23], p. 106).

Certain approaches restricted their focus to specific types of co-design collaborations, for example, between academic researchers and end-user organisations [28] or to collect research data [29]. Approaches also differed in the extent to which they emphasised intensity of engagement. For example, consumer involvement in research regarded co-design as including consultation [10] but approaches such as research engagement stressed the need for “*significant engagement*” [24]. From a regional perspective, we note that ‘patient and public involvement’ was more frequently used for research conducted in the United Kingdom, while ‘research engagement’ was more frequently used for research conducted in the United States.

The co-design approaches identified also differed in the extent to which they were used as umbrella concepts that might contain other co-design approaches [7, 24, 31]. For example, participatory research is described as “*an umbrella term to include all partnered research, including community-based participatory research (CBPR), action research, participatory action research, participatory evaluation, community engagement and patient engagement*” ([15], p. 2).

Several research co-design approaches included overlapping and/or nested conceptual frameworks. These nested conceptual frameworks classified involvement in different ways. For example, patient and public involvement research differentiates between three levels of involvement: “*(1) consultation (where researchers seek the views of the public on key aspects of the research); (2) collaboration (an on-going partnership between researchers and the public throughout the research process)* [and]; *(3) ‘publicly led’ (where the public designs and undertakes the research and where researchers are only invited to participate at the invitation of the public)*” ([23], p. 106). In contrast, the consumer involvement in research approach categorises engagement into eight categories based on the interaction between four levels of researcher and end-user engagement control within a research project [10].

Research co-design approaches varied in the research phase that they considered as relevant to end-user input. For example, participatory methods research appears to focus on involvement in the design and conduct of research [11]. In contrast, stakeholder engagement includes the “*interpretation of the findings, crafting of the message and dissemination of the results*” ([24], p. 1391) while patient engagement includes “*applying* […] *resulting knowledge*” ([7], p. 2). No approaches specifically focused on the research planning phase. Some of the approaches that we identified, such as patient and stakeholder engagement, consumer engagement, and stakeholder involvement were not consistently defined within the literature examined and therefore difficult to understand, compare and differentiate.

### What activities do these research co-design approaches involve?

Our review identified several types of activities involved in research co-design. Most frequently mentioned were activities where research end-user contributions were solicited such as interviews and participation in advisory councils [5]. There were no consistent differences in the activities described by higher-quality (AMSTAR ≥5) compared to lower-quality or non-systematic reviews.

#### Objectives

Co-design activities were typically used to facilitate (1) prioritisation and research agenda-setting [5, 10, 12]; (2) review of research proposals [8, 29] and (3) contribution to study design, outcomes and materials [33]. For example, Schilling and Gerhardus [12] describe a prioritisation process that included an initial survey of older adults with dementia followed by a group workshop. In one review, Yoshida et al. [13] investigated a specific method for prioritisation, exploring how a research prioritisation technique designed to incorporate stakeholder contributions, the Child Health and Nutrition Research Institute method, had been used. Cukor [29] described how a community advisory board drawn from patients with kidney disease and their caregivers reviewed and provided feedback on initial proposals (i.e. research questions) for funded research. Many reviews described structured input from end-users on research protocols, especially in selecting outcome measures that mattered to patients, providing feedback on technical or culturally appropriate language in study materials and operational processes such as recruitment and obtaining consent [12, 31, 34].

#### Types of activity

Foundational co-design activities were typically taken by the researchers prior to any contribution activities, and included identifying end-user groups, defining specific roles and responsibilities for end-users, and recruiting and managing end-users to the research project or programme [33, 35]. The most typical co-design activities followed these foundational activities and were identified by Domecq et al. [5] as focus groups, interviews, surveys and deliberative methods (e.g. rating processes). Across all reviews, group or individual meetings with end-users were most common. Less frequently mentioned activities included telephone calls, citizens’ juries, town meetings, symposia, workshops, conferences, forums, voting, the nominal group technique, one-time priority questionnaires and the Delphi technique [14].

#### Frequency and intensity of engagement

The frequency and intensity of research co-design activities varied greatly across the included reviews – from seeking occasional contributions from end-users to seeking daily involvement and from relatively low-intensity activities such as seeking end-user feedback on research materials to intensely collaborative power-sharing arrangements in end-user-led research (e.g. [10, 23, 29]). Oliver et al. ([10], summary on p. 96) provide a particularly detailed examination of how different activities performed across different levels of researcher and end-user engagement.

#### Roles

Many different roles for co-design participants were described. These included, for example, advisors and committee members who provide advice, reviewers who examined plans and materials, and co-production roles where the co-designers initiated, and/or shared control of the research [33].

#### Tailoring of activities

Co-design activities were tailored to the end-user groups (typically patients) involved. In particular, different activities were recommended for co-design with disabled individuals, children and older patients (e.g. [11, 31]) compared to the general patient population. Though reviews frequently mentioned practitioners such as carers, nurses and doctors as consumers and end-users, they generally did not discuss how co-design should involve these groups or provide any specifically tailored co-design recommendations.

### What do we know about the effectiveness of research co-design?

We did not identify any comparative studies that experimentally compared the use of research co-design against a more traditional research process in terms of end-user health outcomes or cost-effectiveness. Evaluation typically examined researchers’ and users’ perceptions of benefit, the influence of co-design on the research process (e.g. whether a questionnaire changed), and the time and resourcing required. The findings described did not consistently differ on the basis of review quality.

Reviews that evaluated the impact of research co-design on the research process found mixed results [10, 29, 33, 35]. A range of benefits of co-design were described in the included reviews. Firstly, the research topics, research questions and design of materials were perceived to be more applicable and acceptable to research end-users as a result of co-design (e.g. [7, 35]). For example, Di Lorito et al. found that co-design helped “*to tailor the* [survey] *questions so they can be user-friendly, concrete, specific and relevant for participants*” ([32], p. 678). Camden et al. found that co-design led to “*better identification of relevant questions, credibility of the knowledge produced and application of results adapted to contexts*” ([24], p. 1937).

Secondly, a number of benefits for end-users who participated in research co-design were identified, including positive emotional outcomes as well as increased knowledge about and increased skills for contributing to the research process and managing their medical condition [33]. Many reviews specifically noted positive emotions resulting from participation in co-design, including increased confidence in their daily life [7, 31] and a sense of pride and accomplishment [32]. Finally, our review also provided evidence that research co-design can benefit researchers. For instance, Brett et al. note that research co-design can “*build important links with the community and can help with accessing participants, improving response rates, recruitment from seldom heard groups, development of greater empathy with research subjects and better informed consent based on a more informed participant. There is also evidence that* [Public and Patient Involvement] *PPI can help in the assessment and development of research instruments, improve the timing of interventions and ensure the instruments are more acceptable to the community*” ([33], p. 13).

However, some negative aspects of co-design were also identified, including increased time and the financial resources required to incorporate co-design elements into a research process; tensions between researchers and end-users in decision-making and sacrificing scientific rigor for end-user preferences; and concerns regarding study design [5, 10, 29, 32]. For example, Boote et al. outline a case where “*members of the public involved in the design of the stroke trial recommended that additional outcomes be examined in the research. This led the research team to develop non-validated measures for use in the trial*” ([36], p. 18). Similarly Brett et al. [33] mention a case where clinicians, researchers and service users differed in the extent to which they were willing to trade changes in the rigor of the research process for other outcomes such as the quality of patients’ lives. Furthermore, some end-users experienced their involvement as tokenistic, feeling “*not listened to, frustrated and marginalised*” ([33], p. 13).

Evaluations across all research approaches generally measured the near-term effects of engagement (e.g. better research, as assessed by interpretation, and patient empowerment, using qualitative studies). For instance, public and patient involvement research reported that evaluation used a narrative description (e.g. [33]). Similarly, patient engagement research (e.g. [7, 25]) found that the studies evaluating impact generally used qualitative analyses of small samples. The longer-term effects of co-design, for example, on improved health outcomes, were not examined.

## Discussion

Misalignment between researchers’ aims and research end-user needs is a major cause of research waste [1–3]. The effective implementation of research co-design to reduce this misalignment could therefore have substantial positive impacts, from improving health research processes and outcomes, to improving the function of health systems and the societies that depend on them. This review is, to our knowledge, the first overview of reviews of this eclectic literature and the first review motivated by the needs of health funders, policy-makers and practitioners. The results contribute a short and accessible synthesis of research co-design that outlines the state of the science in research co-design approaches, activities and evaluation.

Many research co-design approaches were identified across the included reviews. These generally focused on some form of research-related engagement with one or many groups of non-research stakeholders. We noted several differences between these approaches, for example, in the extent to which they focused on co-design with specific groups, approaches, research phases or levels of engagement. The lack of a singular consistent conceptualisation of ‘co-design’ made it much more difficult to retrieve and understand the relevant literature as recognised by several authors of the reviews that we examined. For example, Drahota et al. [28] note that one challenging aspect of collaboration research is the lack of standardized terminology and conceptual definitions. Similarly, Camden et al. comment that “*search strategies were limited by the great variety of terms used for each of the terms searched*” ([24], p. 1399).

We identified many activities that are involved in research co-design. Consistent with the variation in co-design terminology and definition, there is extensive variance in the timing, participants and aims of co-design activities. Though it was clear that certain research activities were used frequently, there was insufficient detail to establish what was actually involved (e.g. specific interview procedures or how disparate opinions on study prioritisation were reconciled). Reflecting this, several authors noted a need for better description and explanation (e.g. [31, 35]). For example, Puts et al. state “*while there have been many studies that have used patient engagement, the processes involved and the outcomes have not been well documented*” ([35], p. 395). Similarly, Camden et al. noted *“many studies reported having engaged stakeholders throughout the research process, but in only a few articles were we able to identify specific strategies in each research step*” ([24], p. 1398). This combination of vague terminology and vague description of co-design makes it very difficult for researchers to undertake co-design activities, even if they are motivated (or required) to incorporate co-design into their research.

We also found that the effectiveness of research co-design has rarely been tested empirically or experimentally, but that qualitative evaluations were generally positive. These implied that research co-design can have several benefits for research processes, researchers and practitioners, but also create some negative impacts such as frustration, increased complexity and delays in research progress. The reviews identified expressed concerned with the lack of evaluation of research co-design. As Oliver et al. point out “*The literature was replete with enthusiastic reports and reflections but with little or no detail about public involvement, and often little attempt at objectivity*” ([37], p. 78). Similarly, Morley et al. note that “*this scoping exercise has identified evidence of highly variable levels and types of consumer involvement within and beyond Cochrane, but limited evidence for the impact of most methods and levels of involving people*” ([9], p. 16).

What are the key barriers that need to be overcome for better research co-design evaluation? One barrier is that many of the metrics involved, for example, patient knowledge about research, are quite different to those traditionally measured or valued in health. Health researcher expertise in measuring clinical outcomes may not transfer to measuring end-user experiences, voice, shared power and potential impact on the research process itself. A second barrier is a lack of consistency across co-design metrics. Our review identified a plethora of different short- and long-term evaluation metrics, each purporting to measure benefits and costs to patients and researchers but doing so quite differently. A third barrier is a lack of a clearly accepted causal framework or theory of change that (1) argues for the value of measuring specific instrumental (e.g. research design improvement) and terminal outcomes (e.g. health improvements) and (2) explains and justifies the probable relationships between these metrics (e.g. that better research design leads to better health for these reasons, which in turn leads to improved social outcomes).

Esmail et al. [25] provide a foundation for further work to address these barriers. They suggest approaches for better co-design evaluation, for example, that researchers should develop or choose an evaluative framework or set of criteria prior to research-co design, use predefined, validated tools, and conduct regular or continuous evaluations (ideally involving external evaluators). Additionally, they suggest three categories of measurement – context, process and impact of engagement – and outline a range of assessments that have been performed under each category.

Several researchers emphasised the importance of context when determining the design, impact and implementation of health research (e.g. [25, 33, 37]). For example, as Brett et al. explained, “*Context refers to* […] *the setting for the involvement and the atmosphere/attitude in which it is conducted. The process of involvement can include a number of different things. For example, it could refer to the level of involvement that users have, how they are involved, when they are involved, and what procedures are put in place to improve the likelihood of success* […] *If the context and process is not appropriate then the chances of beneficial impact of patient and public involvement activity appear to diminish*” ([33], p. 46). Additionally, the reviews were clear in suggesting that research co-design should be tailored differently across multiple complex contexts and that no one approach was a panacea; as Oliver et al. noted, “*Different methods had varying degrees of success in a range of contexts*” ([37], p. 78).

Our results clearly demonstrate that research co-design involves a broad range of approaches, which range from low effort and low risk through to the much higher effort research co-production approaches. For example, the low-effort and low-risk approaches, might involve asking stakeholders to review and revise research questions in the data collection process. In contrast, co-production could involve included stakeholders with equal decision-making power and responsibility across the entire research process.

It is important to understand the implications of the range and breadth of these research co-design approaches. Each approach lends itself to different contexts; for instance, the level of stakeholder interest and capability mediates whether they should be engaged in a consultative or partnership arrangement. Each approach also suits different types of evaluation. More discrete and limited collaboration isolates specific processes (e.g. consultation about research questions) and therefore allows for the evaluation of very specific processes. In contrast, the co-production processes involve less discrete patterns and periods of interaction, forcing a more aggregated, and infrequent, approach to evaluation. This, in turn, has implications for the comparability of these evaluations and the co-design approaches that they measure.

It is important to link our synthesis of reviews to the related non-review and non-health literature. There are many relevant examples of this literature, including reports explaining how to engage in co-design [19, 38–42], how co-production techniques have been used in applied contexts [43], and research into co-design in non-health contexts [44, 45]. Though these works differ in focus from our overview of reviews, they generally accord with the findings of this study, for example, in recommending research evaluation [39] and flexibility [43]. They also complement our review by providing detailed recommendations for specific bodies [39] and extended instruction on how to use co-design in practice [19, 38–40].

### Recommendations for research

This review has highlighted a number of opportunities for researchers to improve how they implement, evaluate and evolve research co-design. Several reviews recommended the use of more clear and consistent co-design terminology [5, 7]. For example, Domecq et al. notes “*Several authors* [...] *confirmed a need to have clear, consistent terminology to denote patient engagement, which can be used and applied across various contexts to inform a clear conceptualisation and understanding of patient engagement across the research process*” ([5], p. 4). Drahota et al. argue that “*strengthening conceptual clarity by using standardized terminology, definitions, and methods is an important research direction for this field*” ([28], p. 195). Manafo et al. argue that this “*lack of consistency in terminology use and definitions only further adds to the confusion and complexity surrounding patient engagement in research, while diluting the possibility of achieving meaningful and successful engagement from all stakeholders*” ([7], p. 8).

Similarly, better reporting of the activities involved in research co-design is also recommended (e.g. [11, 35]). Some reporting standards have been developed, for example, the use of the Guidance for Reporting Involvement of Patients and Public checklist [46, 47]. As stated by Domecq et al., “*Building a robust patient engagement enterprise requires a firmer and more widespread understanding by both researchers and patients of the ‘how’ to effectively and efficiently include patients in a meaningful and feasible way*” ([5], p. 4).

We see considerable value in greater synthesis and differentiation between the many different strands of research co-design that exist in the literature. Researchers need to understand the underlying differences between approaches in order to (1) understand their relative merits, (2) the applicability/utility of traditional approaches/metrics for evaluating them, and (3) the methodological and theoretical barriers to their systematic comparison using traditional means. Additionally, there is a second continuum of interest – the level of engagement. This ranges from the relatively simple process of engaging stakeholders in decisions about the questions asked and methods for asking them to participate in challenging, messy and unpredictable research co-production. We therefore encourage researchers to build on established accepted typologies and conceptual hierarchies to capture and represent the range of existing co-design approaches and ideally make these more parsimonious. One example of such an approach is the ECOUTER methodology [48].

Perhaps most pressingly, there is a need for much better evaluation of research co-design (e.g. [10, 34, 36]). Esmail et al. accurately highlight the importance of evaluation when they comment that “*the most striking observation* [from our findings] *is how few studies actually assess or formally evaluate any measures of engagement*” ([25], p. 136). Domecq et al. underscore this point when they state that “*this fledgling initiative can ill afford a lack of robust evidence that underlies the impetus supporting patient engagement in research*” ([5], p. 4). It is unsurprising that there is a dearth of studies comparing co-design with ‘business as usual’ or evaluating the real-world impact of co-design (outside of evaluation of satisfaction with the co-design process). The resourcing and logistics requirements of such an endeavour would be significant, for example, requiring comparison of similar research projects with and without co-design and/or long-term (i.e. 3–5 year) follow up of real-world impacts. Whilst the complexity of testing the effectiveness of co-design is acknowledged, this is a critical gap in the literature given the significant investment that is being made in co-design.

One approach to deal with the challenge of evaluating the real-world impact of co-design may be to develop a theory of change linking proximal and easily measurable patient and research outcomes to more diffuse terminal health and social impacts. If doing this, researchers may benefit from adopting ideas from evaluation research (e.g. [49, 50]). For example, Mark and Henry [50] examine and link three levels of analysis (individual, interpersonal and collective) to four categories of change (general influence, cognitive and affective, motivational, and behavioural). Systems thinking and related research may also help with identifying and standardising the understanding of how different metrics interact (e.g. [51, 52]).

There is a need to further explore two relatively fundamental questions: ‘When is it worth it to engage end-users?’ and ‘What are the ideal activities to use in specific contexts?’ The research examined frequently discussed the benefits and costs of co-design to patients and researchers. Some reviews (e.g. [10, 33]) also described individual cases of co-design in some detail and provided useful conceptual frameworks for categorising them. However, no research took a broadly comparative approach to examine, for example, the types of co-design that produce the best outcomes for all parties and for specific parties in particular settings. Additionally, it remains unclear where intensive collaborations and activities are most effective and acceptable.

There is a need for more research to examine how activities should be tailored to particular contexts and end-user groups. For example, the optimal approach to co-design is likely to differ between patients and practitioners. Although there was some discussion of how to use age- or intellect-appropriate co-design activities, the rules of thumb and approaches for tailoring were not addressed. There was virtually no discussion of how to conduct co-design activities with practitioner groups such as doctors, nurses and carers.

Research into the effect of co-design participant group size, and how to tailor to different group sizes, is also needed. Though the research examined frequently mentioned the details of groups involved in co-design, we did not identify any significant discussion of the effect of group size on different co-design activities or the ideal group size for specific contexts.

Future research should explore what types of co-design activities are best suited to specific aspects of the research process. This review focused on the study planning phase as it is particularly critical for avoiding research waste. However, different approaches will be optimal across different phases of the research process – what is most effective for promoting research dissemination is likely to be very different from the best approach to seek survey feedback.

Robust and consistent reporting of research co-design activities, costs and outcomes is a necessary foundation for enhancing our understanding of its effectiveness and cost-effectiveness. Long-term evaluations of co-design to address this gap are therefore required.

### Recommendations for practice

Those engaged in research co-design should use our review to understand which broad concepts are relevant, for example, if searching for relevant literature or case studies. There are a range of relevant terms, such as ‘participatory research’, that practitioners may not expect to relate to research co-design. Other researchers who are new to co-design, and to research engagement generally, should consider how involving research end-users in the study planning phase can assist in prioritising research topics, setting research agendas, reviewing study plans, and in helping to refine research design and processes. Our discussion of research and engagement processes, and our recommendations, should help practitioners to better plan and execute co-design by highlighting issues to expect and prepare for as well as by providing templates to learn from and follow. Our discussion of the evaluations of research co-design, particularly the costs and benefits identified, may be useful for practitioners who are evaluating whether research co-design is appropriate for a research project that they seek to fund or conduct.

Defining best practice in research co-design on the basis of this rapid overview of reviews is difficult due to the breadth of co-design activities, myriad opportunities across the research planning phase, and the lack of comprehensive or conclusive evaluations regarding the impact of co-design activities on research outcomes. In addition, some studies highlighted tensions between researcher and end-user incentives and preferences (e.g. [33]), leading to compromised study designs, experiences of tokenism, and/or frustration and disappointment at the missed co-design opportunity. The specific health context, end-user readiness for contribution, and researcher capability will vary widely between research projects. In a review of Australian research co-design attempts, Miller et al. wrote that “*the effectiveness of strategies used in consumer and community engagement in health and medical research is highly context-specific, and in many instances dependent on the attitudes, skill, and relationships between the consumers and researchers involved in the research process*” ([26], p. 3).

Several papers mention that co-design projects often face significant challenges in instigating and maintaining co-design-related collaboration (e.g. 33). As this is in part a behavioural problem, we recommend that researchers draw on behavioural insights where relevant. For example, the Fogg Behaviour Model [53] or the COM-B Behaviour Change model [54] could be used to diagnose actors’ capabilities, motivation, and triggers or opportunities. Similarly, the EAST model can improve end-user engagement by designing activities and communications that are easy, attractive, social and timely to respond to/participate in [55].

We have also organised and synthesised common recommendations from the rapid overview of reviews (Additional file 5), using the abbreviation INVVOLVE. These are outlined in Table 2. Readers should note that this table is not proposed as an approach to research co-design, a census of all recommendations, or a set of steps to follow in sequence.

**Table 1:** Examples of research co-design approaches identified

Approach	Definition (reference)
Patient and public involvement	“*Doing research ‘with’ or ‘by’ the public, rather than ‘to’, ‘about’ or ‘for’ the public*” ([23], p. 106)
Stakeholder engagement	“*Significant collaboration with knowledge users, including the development or refinement of the research questions, selection of the methodology, data collection and tools development, selection of outcome measures, interpretation of the findings, crafting of the message and dissemination of the results*” ([24], p. 1391)
Participatory research	“*We use PR as an umbrella term to include all partnered research, including community-based participatory research (CBPR), action research, participatory action research, participatory evaluation, community engagement and patient engagement), and community engagement continue to attract increased attention as an approach to research, requiring formation of teams of researchers in partnerships with those affected by the issue under study in the community and those who will utilize the results to effect change*” ([15], p. 1)
Patient and stakeholder engagement	Not defined [25]
Consumer engagement	Not defined [26]
Participatory methods	“*Any method that can be used to obtain children’s views, aiming to involve them in the design and conduct of research*” ([11], p. 682)
Inclusive health research	“*Research which includes or involves people with learning disabilities as more than just subjects of research*” ([27], p. 275)
Community- academic partnership	“*The collaboration must have been between at least one academic partner (e.g. investigator(s) in a university department, university hospital, university medical center) and at least one community organization or stakeholder (e.g. community agency, church, school, policy-maker, according to a definition adopted in order to maximise the number of articles eligible for inclusion), and have shown some indication of shared control or shared decision making, as described in the collaboration’s collaborative or specific actions*” ([28], p. 169)
Community- based participatory research	“*A collaborative approach to research that equitably involves all partners in the research process and recognizes the unique strengths that each brings*” ([29], p. 1703)
Stakeholder involvement	Not defined [13]
Patient engagement	“*Occur*[ing] *when patients meaningfully and actively collaborate in the governance, priority setting, and conduct of research, as well as in summarizing, distributing, sharing, and applying its resulting knowledge*” ([7], p. 2)
Consumer involvement in research	Consumers defined as: “*Users and potential users of services, products and resources (including natural resources). In health this includes patients and potential patients; long-term users of services; carers and parents; organisations that represent consumers’ interests; members of the public who are the targets of health promotion programmes; and groups asking for research because they believe they have been exposed to potentially harmful circumstances, products or services. Depending on the context, consumers may also be described with any of the following terms: ‘lay’, ‘non-expert’, ‘service user’, ‘survivor’ or ‘member of the general public*” Involvement defined as “*any form of participation in the making of decisions, at whatever stage or level, from consultation at the end of the decision-making process to joint working throughout the entire decision-making process*” ([10], p. vii)

### Limitations

Rapid reviews, including rapid overview of reviews, are, by nature, more rapid than other types of research synthesis and can therefore lack the detail that traditional systematic reviews provide [16]. However, despite these issues, rapid reviews and systematic reviews generally result in similar conclusions [56]. Nonetheless, a more detailed and thorough review might yield different insights and conclusions.

Our original intention was to focus on review-level literature examining the planning phase of research. However, we found that this literature encompassed a broader definition than ours, because the primary studies within these reviews used co-design across all of the different research phases. These reviews, their findings and our discussion of these findings therefore include consideration of types of co-design and phases of the research cycle outside the research planning phase that was the focus of this review (i.e. co-design up to the point of finalising the research question and design). This was unavoidable as a more restrictive search limited to reviews that solely synthesised research from primary studies that examined the research planning phase would have resulted in no studies to include or useful insights to share. When analysing the review-level literature, we identified the co-design principles that were most relevant to the research development phase of research. However, most reviews did not categorise co-design principles by research phases and the diversity, and variable use, of terms used to refer to research co-design further complicated the task as it was difficult to distinguish between different descriptions of phases. Despite this limitation, the co-design principles elucidated by the review are phase agnostic and thus applicable and useful for guiding co-design within the critical research planning and design phase.

Research co-design is a composite of research and practice and conceptually non-homogeneous across the many contexts in which it is used. Accordingly, our use of a systematic review of systematic reviews was less effective than in most cases as this approach assumes the conceptual consistency of elements within the reviews included.

Because we restricted our search to reviews in English, we may have omitted relevant research written in other languages. Because we conducted an overview of reviews, the design of our study excluded all relevant primary literature. We only had one quality appraiser; this was necessary due to the resourcing available to conduct the review. We only conducted quality appraisal on systematic reviews as the AMSTAR tool that we used was not compatible with non-systematic reviews and we were not aware of tools for assessing narrative reviews [57]. We therefore cannot attest to the quality of the non-systematic reviews included in this study.

## Conclusion

Research co-design appears to be widely used but seldom described consistently or evaluated in detail. Though it has rarely been tested empirically or experimentally, existing research suggests that it can benefit researchers, practitioners, research processes and research outcomes. Realising the potential of research co-design may require the development of clearer and more consistent terminology, better reporting of research activities, and long-term evaluation of co-design outcomes and impact.

## Supplementary information

**Additional file 1:** Deviations from Protocol

**Additional file 2:** Search terms and search strings

**Additional file 3:** Included records

**Additional file 4:** Records excluded at full text screening

**Additional file 5:** Extraction tables

**Additional file 6:** Quality assessment

## Data Availability

The data supporting the conclusions of this article is included within the article and its supplementary files.

## Abbreviations

AMSTAR: Assessing the Methodological Quality of Systematic Reviews
PCORI: Patient Centred Outcomes Research Institute
PRISMA: Preferred Reporting Items for Systematic Reviews and Meta-Analyses
