# Supplementary material for: Research co-design in health: a rapid overview of reviews
Source: Health Res Policy Syst. 2020 Feb 11;18:17. doi: 10.1186/s12961-020-0528-9 (PMC7014755; doi:10.1186/s12961-020-0528-9)
Supplement: Supplementary file 1 — Additional file 1: Deviations from Protocol [file 12961_2020_528_MOESM1_ESM.docx]

Additional File 1: Deviations from protocol

We said that we “will seek extra information about where to find additional studies from known networks and experts in the field. After all searches have been completed, we will search Google Scholar for relevant studies that have been omitted from our other searches.”

We did not perform these additional searches for reasons of feasibility. Our initial yield was larger than expected. Additionally, we had to individually review and download records from each of the three grey databases examined, which was considerably more time consuming than expected.

We updated our research questions in response to feedback from reviewers.
