# Supplementary material for: Research co-design in health: a rapid overview of reviews
Source: Health Res Policy Syst. 2020 Feb 11;18:17. doi: 10.1186/s12961-020-0528-9 (PMC7014755; doi:10.1186/s12961-020-0528-9)
Supplement: Supplementary file 2 — Additional file 2: Search terms and search strings [file 12961_2020_528_MOESM2_ESM.docx]

Additional file 2: Search terms and search strings

# Search terms

| **Concept** | **Sub-concept** | **Search terms derived** |
| --- | --- | --- |
| Actors (Who) | Patient | Patient, User, Surrogate, Advocate, Client |
|  | Clinician | Clinician, Doctor, Provider |
|  | Funder | Funder, Agency |
|  | Carer | Carer, Caregiver, Relative |
|  | Payer | Payer, Purchaser, Insurer, Employer, Pharmaceutical, Hospital, "health system" |
|  | Generic stakeholder | Public, Community, Stakeholder, Consumer, Lay person |
| Research co-design processes  & engagement points | Involvement types (WHAT) | Consult, Collaborate, Input, Engage, Involve, Design, Produce, Codesign, Coproduce, Partner, Participate, Voice, Dialog, Opinion, Develop |
|  | [engagement] Processes (HOW) | Process, Logic, Framework, Guidelines, Guidance, Patient satisfaction, Needs assessment, Delphi, Interview, Focus group, Advisory board |
|  | Engagement points (WHEN) | Design, Planning, Decision, Develop, Study outcomes, Research question, Research aims |
| Qualifiers (where) | Research in health rather than health care design or preferences | Research, Trial, Study, Outcome |

# Search strings

## MEDLINE (Ovid)

http://ovidsp.ovid.com/ovidweb.cgi

|  |  |
| --- | --- |
| Search # | String |
| 1 | (Patient* or User* or Clinician* or Provider* or Caregiver* or Public or Communit* or Stakeholder* or Consumer* or Client*).tw. |
| 2 | (Collaborat* or Consult* or Engag* or Involv* or Design* or Participat* or Partner* or Develop* or "co-design" or "co design" or "codesign").tw. |
| 3 | exp community-based participatory research/ or exp Patient Participation/ or exp Community Participation/ |
| 4 | ((Patient* or User* or Clinician* or Provider* or Caregiver* or Public or Communit* or Stakeholder* or Consumer*) adj2 (Collaborat* or Consult* or Engag* or Involv* or Design* or Participat* or Partner* or Develop* or "co-design" or "co design" or "codesign")).tw. |
| 5 | (3 or 4) and ((research* or study or trial or outcome*).ti. or research design/) |
| 6 | ("29796308" or "24783309" or "28625273" or "28120462" or "26496124" or "26766577" or "25885588" or "25890601" or "25582284" or "25323964" or "25034612" or "25047393" or "24893581" or "24581296" or "24568690" or "23390509" or "23618406" or "23318684" or "22853705" or "22353031" or "22109631" or "21641075" or "26689460").ui. |
| 7 | limit 5 to "review articles" |

## Cochrane Database of Systematic Reviews

https://www.cochranelibrary.com

|  |  |
| --- | --- |
| ID | Search |
| #1 | MeSH descriptor: [Community-Based Participatory Research] explode all trees |
| #2 | MeSH descriptor: [Stakeholder Participation] explode all trees |
| #3 | MeSH descriptor: [Community Participation] explode all trees |
| #4 | MeSH descriptor: [Patient Participation] explode all trees |
| #5 | (Research or Trial or Study or Outcome*):ti |
| #6 | ((Patient* OR User* OR Clinician* OR Provider* OR Caregiver* OR Public OR Communit* OR Stakeholder* OR Consumer*) NEAR (Collaborat* OR Consult* OR Engag* OR Involv* OR Design* OR Participat* OR Partner* OR Develop* OR "co-design" OR "co design" OR "codesign")):ti,ab,kw |
| #7 | #5 and (#1 or #2 or #3 or #4 or #6) in Cochrane Reviews |

#

# PSYCINFO (Ovid)

http://ovidsp.ovid.com/ovidweb.cgi

|  | OVID PSYCINFO |
| --- | --- |
| Search # | String |
| 1 | (Patient* or User* or Clinician* or Provider* or Caregiver* or Public or Communit* or Stakeholder* or Consumer* or Client*).tw. |
| 2 | (Collaborat* or Consult* or Engag* or Involv* or Design* or Participat* or Partner* or Develop* or "co-design" or "co design" or "codesign").tw. |
| 3 | exp Community Involvement/ or exp Client Participation/ or exp Community Participation/ |
| 4 | ((Patient* or User* or Clinician* or Provider* or Caregiver* or Public or Communit* or Stakeholder* or Consumer*) adj2 (Collaborat* or Consult* or Engag* or Involv* or Design* or Participat* or Partner* or Develop* or "co-design" or "co design" or "codesign")).tw. |
| 5 | (3 or 4) and ((research* or study or trial or outcome*).ti. or research design/) |
| 6 | limit 5 to ("0800literature review" or "0830systematic review") |
