# Supplementary material for: Research co-design in health: a rapid overview of reviews
Source: Health Res Policy Syst. 2020 Feb 11;18:17. doi: 10.1186/s12961-020-0528-9 (PMC7014755; doi:10.1186/s12961-020-0528-9)
Supplement: Supplementary file 3 — Additional file 3: Included records [file 12961_2020_528_MOESM3_ESM.docx]

Additional File 3: Included Records

# Included records

|  | **Research areas for co-design mentioned** | **Studies included** | **Study designs of studies included in review:** |
| --- | --- | --- | --- |
| Guise et al., 2013 | stakeholder engagement | 56 | Not reported |
| Domecq et al., 2014 | patient engagement | 142 | 8 systematic reviews, 7 RCTs, 103 qualitative studies, 8 single cohort, 9 cross-sectional, 7 case reports |
| Salsberg et al., 2015 | participatory research | 54 | Not reported |
| Esmail et al., 2015 | patient and stakeholder engagement | 108 | Not reported |
| Brett et al., 2014 | Public and patient involvement (PPI) | 66 | two were randomized‐controlled trials (RCTs), one was a pre‐test/post‐test study, one was a cohort, 46 were qualitative studies, nine were cross‐sectional, five were case studies and two were case series |
| Miller et al., 2017 | consumer engagement | 168 | Not reported |
| Haijes & van Thiel, 2016 | participatory methods | 24 | 11 reviews, 1 research proposal, and 2 research papers initally. After hand search of the bibliography of included papers, added 3 additional reviews, 7 original research papers, 1 book chapter, and 2 guidelines |
| Frankena et al., 2015 | inclusive health research | 26 | Not reported |
| Drahota et al., 2016 | community-academic partnership | 50 | Case studies |
| Cukor et al., 2016 | community-based participatory research | 7 | Not reported |
| Yoshida et al., 2016 | stakeholder involvement | 12 | Not reported |
| Puts et al., 2017 | patient engagement | Not reported | Not reported |
| Manafo et al., 2018 | patient engagement |  | Case study/series (n = 7)  Experimental (n = 1)  Quasi-Experimental (n = 2)  Non-experimental (n = 15)  Qualitative interviews (n = 7)  Literature review (n = 11)  Commentary/Editorial (n = 1)  (n = 44, formal review; n = 11, informal review/grey literature) |
| Schilling & Gerhardus, 2017 | patient and public involvement | 9 | Not described |
| Bailey et al., 2015 | Public and patient involvement (PPI) |  | seven review articles, eight original research papers, three reports, three guidelines and one webpage |
| Camden et al., 2015 | stakeholder engagement | 19 | Not reported |
| Lee et al., 2017 | Patient engagement | 3 | Not reported |
| Di Lorito et al., 2018 | Co-research | 13 | feasibility study (1), case reports (12) |
| Fudge et al., 2007 | user involvement | 30 (35 papers) | Not reported |
| Boote et al., 2010 | Public involvement | 7 | Primary studies |
| Oliver et al., 2004 | Consumer involvement | 135 | 13 Type A: Research programmes inviting the collaboration of consumer groups  14 Type B: Research programmes consulting consumer groups  5 Type C: Research programmes inviting the collaboration of individual consumers  13 Type D: Research programmes consulting individual consumers  12 Type E: Research programmes responding to consumer action with collaboration  2 Type F: Research programmes responding to consumer action with consultation  7 Type G: Research programmes tapping consumer perspectives indirectly  7 Type H: Consumer-led research programmes |
| Boote et al., 2011 | Public involvement | 7 | Not specified |
| Oliver et al., 2008 | Public involvement | Not specified | Not specified |
| Boote et al., 2012 | Public involvement | 9 | Not specified |
| Morley et al., 2016 | Consumer involvement | 36 | Organisational level evaluations, CRG case reports, Individual reviews, Syntheses/summaries |
| Brett et al., 2010 | patient and public involvement | 83 published, 8 grey literature | 2 were randomised controlled trials (RCTs), 52 were qualitative studies, 15 were case studies or case series, 4 were cross-sectional studies, and 10 were structured reviews. |
