# Supplementary material for: Research co-design in health: a rapid overview of reviews
Source: Health Res Policy Syst. 2020 Feb 11;18:17. doi: 10.1186/s12961-020-0528-9 (PMC7014755; doi:10.1186/s12961-020-0528-9)
Supplement: Supplementary file 5 — Additional file 5: Extraction tables [file 12961_2020_528_MOESM5_ESM.docx]

Additional file 5: Extraction Tables

[Guise et al., 2013: A practice-based tool for engaging stakeholders in future research: a synthesis of current practices. 2](#_Toc12638189)

[Domecq et al., 2014: Patient engagement in research: a systematic review. 3](#_Toc12638190)

[Salsberg et al., 2015: Successful strategies to engage research partners for translating evidence into action in community health: a critical review. 4](#_Toc12638191)

[Esmail et al., 2015: Evaluating patient and stakeholder engagement in research: moving from theory to practice. 5](#_Toc12638192)

[Brett et al., 2014: Mapping the impact of patient and public involvement on health and social care research: a systematic review. 6](#_Toc12638193)

[Miller et al., 2017: Integrating consumer engagement in health and medical research - an Australian framework. 7](#_Toc12638194)

[Haijes & van Thiel, 2016: Participatory methods in pediatric participatory research: a systematic review. 8](#_Toc12638195)

[Frankena et al., 2015: Active involvement of people with intellectual disabilities in health research - A structured literature review. 9](#_Toc12638196)

[Drahota et al., 2016: Community-Academic Partnerships: A Systematic Review of the State of the Literature and Recommendations for Future Research. 10](#_Toc12638197)

[Cukor et al., 2016: Patient and Other Stakeholder Engagement in Patient-Centered Outcomes Research Institute Funded Studies of Patients with Kidney Diseases. 11](#_Toc12638198)

[Yoshida et al., 2016: Setting health research priorities using the CHNRI method: III. Involving stakeholders. 12](#_Toc12638199)

[Puts et al., 2017: Patient engagement in research with older adults with cancer. 13](#_Toc12638200)

[Manafo et al., 2018: Patient engagement in Canada: a scoping review of the 'how' and 'what' of patient engagement in health research. 14](#_Toc12638201)

[Schilling & Gerhardus, 2017: Methods for Involving Older People in Health Research—A Review of the Literature 15](#_Toc12638202)

[Bailey et al., 2015: Involving disabled children and young people as partners in research: a systematic review. 16](#_Toc12638203)

[Camden et al., 2015: Engaging stakeholders in rehabilitation research: a scoping review of strategies used in partnerships and evaluation of impacts. 18](#_Toc12638204)

[Lee et al., 2017: Patient engagement in the design and execution of urologic oncology research. 20](#_Toc12638205)

[Di Lorito et al., 2018: Co-research with adults with intellectual disability: A systematic review. 21](#_Toc12638206)

[Fudge et al., 2007: Involving older people in health research. 22](#_Toc12638207)

[Boote et al., 2010: Public involvement at the design stage of primary health research: a narrative review of case examples. 23](#_Toc12638208)

[Oliver et al., 2004: Involving consumers in research and development agenda setting for the NHS: developing an evidence-based approach. 24](#_Toc12638209)

[Boote et al., 2011: Public involvement in the systematic review process in health and social care: a narrative review of case examples. 25](#_Toc12638210)

[Oliver et al., 2008: A multidimensional conceptual framework for analysing public involvement in health services research. 26](#_Toc12638211)

[Boote et al., 2012: Involving the public in systematic reviews: a narrative review of organizational approaches and eight case examples 27](#_Toc12638212)

[Morley et al., 2016: A systematic scoping review of the evidence for consumer involvement in organisations undertaking systematic reviews: focus on Cochrane 28](#_Toc12638213)

[Brett et al., 2010: The PIRICOM Study: A systematic review of the conceptualisation, measurement, impact and outcomes of patients and public involvement in health and social care research 29](#_Toc12638214)

# Guise et al., 2013: A practice-based tool for engaging stakeholders in future research: a synthesis of current practices.

| Author(s) | Guise, Jeanne-Marie; O'Haire, Christen; McPheeters, Melissa; Most, Carole; Labrant, Lia; Lee, Kathy; Barth Cottrell, Erika K; Graham, Elaine |
| --- | --- |
| Year | 2013 |
| Title | A practice-based tool for engaging stakeholders in future research: a synthesis of current practices. |
| Source | Journal of clinical epidemiology |
| DOI | 10.1016/j.jclinepi.2012.12.010 |
| AMSTAR (/11) | NONE |
| RQ 1: What research co-design processes exist in health settings? | |
| Stakeholder engagement. | |
| RQ 2: What do these research co-design processes involve? | |
| One-to-one (in-person or telephone) calls, focus groups, citizens’ juries, town meetings, symposia, workshops, conferences, forums, consensus, voting, the nominal group technique, one-time priority questionnaires and the Delphi technique | |
| RQ 3: What do we know about the effectiveness of existing research co-design processes? | |
|  | |
| Recommendations | |
| Several recommendations made (see page 671):  Avoid these barriers for effective stakeholder engagement:  Lack of time on the part of stakeholders (busy)  Lack of release time and compensation for members of the public  Researcher need for quick response (time frame too short for community to weigh in)  Stakeholder needs not met in previous engagement  Pursue these enablers for effective stakeholder engagement:  Engage stakeholders early in the process  Before engagement, clearly detail expectations (e.g., anticipated commitment of time and types of activities)  Maintaining ongoing relationships is important to building trust and credibility  Provide opportunities for people to ask questions before meetings (particularly for consumers or others who are unfamiliar with research)  Premeeting information materials promote comfort with the topic and enable informed discussion  Premeeting ‘‘icebreakers,’’ especially when engaging stakeholders with differing experiences/perspectives  Having someone with similar training as the stakeholder can be helpful particularly thought to be important for clinicians  No stakeholder’s comment is considered superior or inferior to another perspective  Follow up presentation of results is important to stakeholders  Be clear about the stakeholder roles  Do not expect community members to do academic duties  Be sensitive to the time constraints of all stakeholders  Use facilitators for stakeholder engagement  Trained/neutral facilitator encourages participation and focuses discussion at in-person meetings  Facilitator [is] knowledgeable of various stakeholder environments (cultural understanding)  Skilled facilitator ensures all stakeholders are heard and manages dominance issues  They also recommend a checklist which outlines a series of recommended activities for conducting research co-design (see p. 673 & 678). | |

# Domecq et al., 2014: Patient engagement in research: a systematic review.

| Author(s) | Domecq, Juan Pablo; Prutsky, Gabriela; Elraiyah, Tarig; Wang, Zhen; Nabhan, Mohammed; Shippee, Nathan; Brito, Juan Pablo; Boehmer, Kasey; Hasan, Rim; Firwana, Belal; Erwin, Patricia; Eton, David; Sloan, Jeff; Montori, Victor; Asi, Noor; Dabrh, Abd Moain Abu; Murad, Mohammad Hassan |
| --- | --- |
| Year | 2014 |
| Title | Patient engagement in research: a systematic review. |
| Source | BMC health services research |
| DOI | 10.1186/1472-6963-14-89 |
| AMSTAR (/11) | 7 |
| RQ 1: What research co-design processes exist in health settings? | |
| Patient engagement | |
| RQ 2: What do these research co-design processes involve? | |
| Focus group, interview, survey, deliberation, and organisational participation. | |
| RQ 3: What do we know about the effectiveness of existing research co-design processes? | |
| Found that most studies reported mainly positive effects of engagement. A smaller number described potential harms or adverse effects that mainly related to patient frustration with the length of the process involved, and aspects like training, transportation, and attendance.Several studies reported that engaging patients in research improves patient enrollment and decreased attrition. | |
| Recommendations | |
| Unable to "recommend best practices on the basis of comparative evidence" (p. 7). The studies that they examined suggested avoiding barriers with logistic and time costs, and scope creep to include irrelevant concerns. These studies also recommended "spending adequate time to build reciprocal relationships [...], fostering mutual respect, and developing clear expectations that are explicitly described and documented in study protocols" (p. 6). | |

# Salsberg et al., 2015: Successful strategies to engage research partners for translating evidence into action in community health: a critical review.

| Author(s) | Salsberg, Jon; Parry, David; Pluye, Pierre; Macridis, Soultana; Herbert, Carol P; Macaulay, Ann C |
| --- | --- |
| Year | 2015 |
| Title | Successful strategies to engage research partners for translating evidence into action in community health: a critical review. |
| Source | Journal of environmental and public health |
| DOI | 10.1155/2015/191856 |
| AMSTAR (/11) | NONE |
| RQ 1: What research co-design processes exist in health settings? | |
| Participatory Research  partnered research  community-based participatory research (CBPR)  action research  participatory action research  participatory evaluation  community engagement  patient engagement | |
| RQ 2: What do these research co-design processes involve? | |
|  | |
| RQ 3: What do we know about the effectiveness of existing research co-design processes? | |
|  | |
| Recommendations | |
| Recommends a process for co-design  "(1) Development of an advisory committee (i) A composition of researchers, the intended users of the research, and/or representatives of community organizations (ii) Advisory committees allow for inclusion of all viewpoints throughout the research process and joint development of dissemination strategies and action plans (iii) Subcommittees are often used to divide up tasks (e.g., reviewing new proposed research topics, articles for publication, partnership evaluation)  (2) Development of research agreements (i) Before the research begins, clearly spell out researchers and partner roles and responsibilities, outline how decisions will be made (e.g., by consensus or by voting), and set out what to do if conflict arises (ii) Research agreements may also include plans for data ownership and control, interpretation of data, and procedures for resolving disagreement over research results (iii) Developing agreements is seen as a trust-building exercise  (3) Use of group facilitation techniques (i) Can be both a formal and an informal process to ensure meaningful involvement and participation of partners (ii) Formal facilitation includes focus groups, workshops, and nominal group techniques (iii) Informal techniques include circulating agendas ahead of time, small group work, and one-on-one informal discussions  (4) Hiring staff from the community of study (i) Hiring local persons as project staff recognizes community members’ abilities to establish good relationships with individual participants for recruitment and ongoing data collection (ii) Projects hire well-respected community members as a “community champions,” field coordinators, intervention staff, interviewers, and group cofacilitators, for data collection and analysis.  (5) Frequent communication (i) Communication between partners through regular group meetings to keep all partners updated on progress and changes in procedures and as a way of discussing concerns and challenges (ii) Other methods include telephone calls to partners who missed meetings to bring them up-to-date and prompt circulation of meeting minutes and newsletters" (p. 10)  See pages 11 & 12 for a table with additional recommendations. | |

# Esmail et al., 2015: Evaluating patient and stakeholder engagement in research: moving from theory to practice.

| Author(s) | Esmail, Laura; Moore, Emily; Rein, Alison |
| --- | --- |
| Year | 2015 |
| Title | Evaluating patient and stakeholder engagement in research: moving from theory to practice. |
| Source | Journal of comparative effectiveness research |
| DOI | 10.2217/cer.14.79 |
| AMSTAR (/11) | NONE |
| RQ 1: What research co-design processes exist in health settings? | |
| patient-engaged research  patient?engagement  stakeholder?engagement | |
| RQ 2: What do these research co-design processes involve? | |
|  | |
| RQ 3: What do we know about the effectiveness of existing research co-design processes? | |
| They find "limited evaluation measures [...] the near-term effects of engagement (e.g., better quality research, patient empowerment). We did not identify any suggested or assessed measures that evaluate the longer-term, downstream effects of engagement, such as improved decision-making or health outcomes." (p.135-136)  "Of the studies that formally evaluated impact, most were qualitative, involving retrospective accounts of engagement experience and relying on self-report through focus groups, one-on-one semistructured interviews, informal observation and/or written surveys with open-ended text responses...The literature most often reported the impact of engagement on those patients engaged, and on the research itself" (p. 136)  Tables 2 & 3 "map the intended purposes of engagement (hypothesized impacts) against those indicators that have actually been developed and measured (suggested and assessed measures" | |
| Recommendations | |
| They make several recommendations for evaluation:  "Establish or select an evaluative framework or set of criteria for patient or stakeholder engagement in research prior to the execution of any research activity (in conjunction with the patients or other stakeholders engaged for collaborative and user-led types of engagement).  • To the extent possible, using predefined, validated tools  • Conducting evaluations at continuous or regularly scheduled intervals throughout the engagement process and, if possible, involving external evaluators  • Documenting the context and process of engagement as fundamental components of the evaluation (i.e., context refers to funding, policy, physical environment or attitudes of those involved; process refers to the level of involvement of users, how they are involved, when, and what procedures are in place to improve likelihood of success)" (p. 137) | |

# Brett et al., 2014: Mapping the impact of patient and public involvement on health and social care research: a systematic review.

| Author(s) | Brett, Jo; Staniszewska, Sophie; Mockford, Carole; Herron-Marx, Sandra; Hughes, John; Tysall, Colin; Suleman, Rashida |
| --- | --- |
| Year | 2014 |
| Title | Mapping the impact of patient and public involvement on health and social care research: a systematic review. |
| Source | Health expectations |
| DOI | 10.1111/j.1369-7625.2012.00795.x |
| AMSTAR (/11) | 7 |
| RQ 1: What research co-design processes exist in health settings? | |
| patient and public involvement | |
| RQ 2: What do these research co-design processes involve? | |
| "Involving mothers of pre-school or primary school aged children to develop topics of research to improve health and wellbeing of families and children before school age; involving stroke patients to identify a research study about awareness and knowledge of stroke and stroke risk and involving mental health users in research on adult mental health services" (p. 640)  "There was also evidence of users being recruited onto steering groups or advisory groups to help advise on research studies, for example, to direct primary health and social care research agenda in one city in the UK, by sitting on a steering group for a randomized control trial of HRT and breast cancer and by sitting on the steering group for research into Paget’s disease. Panels of consumers also helped funders to identify which research proposals should be accepted" (p. 640)  "During the development of the research protocols, users offered pragmatic criticism and commented on the extent to which they perceived the research to be relevant or appropriate to users. Examples included identifying cultural issues that should be taken into account when designing the study identifying patient important outcome measures, solving issues around how to get informed consent and advising on the appropriateness of design from the user perspective.Input from users also helped adapt academic language to suit lay audience, by improving the wording of patient information and invitation letters,and improving the sensitivity of the wording of the information to ensure cultural appropriateness. There was also evidence that users assisted in recruitment by providing greater access to the research community and by identifying the effective ways of accessing participants." (p. 640)  "studies reported that user involvement helped in assessing the appropriateness of research instruments from a communityperspective, in order to develop user-relevant tools and assisted in the development of questionnaire/interview schedules by identifying lines of inquiry not previously considered, helping with the wording of questions, assisting with the timing of interventions and ensuring questions asked were acceptable to the local community. In one study, users helped researchers gain invaluable cultural perspectives of diabetes, particularly how diabetes was often concealed in certain communities because of social stigma, which helped develop a more appropriate study protocol." (p. 640 & 641) | |
| RQ 3: What do we know about the effectiveness of existing research co-design processes? | |
| Found evidence of positive impacts of user involvement, including users helping to:  Identify user?relevant topics for the research agenda  Prioritize topics for the research agenda  Develop the patient?relevant commissioning briefs  Develop user?relevant research questions.  Direct primary health and social care research agendas  Identify which research proposals should be funded  Also, identified some challenging impacts:  Divergence from scientific methods  Ethical dilemmas  Power struggles between researchers and patients and patient groups  Time and cost | |
| Recommendations | |
|  | |

# Miller et al., 2017: Integrating consumer engagement in health and medical research - an Australian framework.

| Author(s) | Miller, Caroline L; Mott, Kathy; Cousins, Michael; Miller, Stephanie; Johnson, Anne; Lawson, Tony; Wesselingh, Steve |
| --- | --- |
| Year | 2017 |
| Title | Integrating consumer engagement in health and medical research - an Australian framework. |
| Source | Health research policy and systems |
| DOI | 10.1186/s12961-017-0171-2 |
| AMSTAR (/11) | NONE |
| RQ 1: What research co-design processes exist in health settings? | |
| Consumer engagement | |
| RQ 2: What do these research co-design processes involve? | |
|  | |
| RQ 3: What do we know about the effectiveness of existing research co-design processes? | |
|  | |
| Recommendations | |
| Argues that four organisational dimensions "contribute to success in consumer and community engagement, namely governance, infrastructure, capacity and advocacy[...].”  Governance  Structures: concerted efforts through the establishment of shared supportive structures.  Policy: comprehensive organisation-wide policy, including acknowledgement of consumers as key stakeholders in all research; partnership roles decided through consultation between consumers, community and researchers, which are based on mutual respect for one another’s different knowledge and experience; and resources including a practice guide to support policy implementation Research funding: ensuring that consumers have an influential and sustained voice in research funding decisions.  Infrastructure  Consumer registers: registers of consumers, with experience working in research, advocacy and policy development, interested in research decision-making and support.  Information: formal and informal support networks and resources and opportunity for consumers to share information and advice.  Capacity  Consumer training: adequate support through training, education and resources appropriate to the expected roles.  Researcher training: program for researchers to better understand the contribution that the community can make to the research as active partners.  Advocacy  As a research organisation, actively promoting and advocating for greater consumer participation in health and medical research" (p. 3 & 4) | |

# Haijes & van Thiel, 2016: Participatory methods in pediatric participatory research: a systematic review.

| Author(s) | Haijes, Hanneke A; van Thiel, Ghislaine J M W |
| --- | --- |
| Year | 2016 |
| Title | Participatory methods in pediatric participatory research: a systematic review. |
| Source | Pediatric research |
| DOI | 10.1038/pr.2015.279 |
| AMSTAR (/11) | 3 |
| RQ 1: What research co-design processes exist in health settings? | |
| participatory methods | |
| RQ 2: What do these research co-design processes involve? | |
| Observational Methods where activities and behaviours are recorded  Verbal Methods such as interviews, and focus groups  Written Methods, such as essays and questionnaires  Visual Methods such as drawings and video diaries  Active Methods, including playing with puppets  Refer to Tables 2 & 3 for more detail. | |
| RQ 3: What do we know about the effectiveness of existing research co-design processes? | |
|  | |
| Recommendations | |
| Recommend a generally applicable approach for determining when to do research co-design: "According to Smith et al. (9), four questions need to be answered prior to involve children in research: “can we make participatory research with children and young people work, will the results be accepted as legitimate findings, should we undertake this kind of research, and is it worth the additional demands on time, resources, and expertise?" (p. 681)  Provides a range of recommendations for co-design with children. See pages 680 and 681. | |

# Frankena et al., 2015: Active involvement of people with intellectual disabilities in health research - A structured literature review.

| Author(s) | Frankena, Tessa Kim; Naaldenberg, Jenneken; Cardol, Mieke; Linehan, Christine; van Schrojenstein Lantman-de Valk, Henny |
| --- | --- |
| Year | 2015 |
| Title | Active involvement of people with intellectual disabilities in health research - A structured literature review. |
| Source | Research in developmental disabilities |
| DOI | 10.1016/j.ridd.2015.08.004 |
| AMSTAR (/11) | NONE |
| RQ 1: What research co-design processes exist in health settings? | |
| inclusive health research | |
| RQ 2: What do these research co-design processes involve? | |
| Involving patients in:  -Research partnerships  -Deciding on the research topic, questions and methods.  -Applying for ethical approval.  -Reviewing literature.  -Developing accessible materials.  -Recruiting participants with [Intellectual Disability]  -Designing and conducting questionnaires.  -Designing and conducting interviews.  -Designing and moderating focus groups.  Methods such as questionnaires, focus groups, and interviews. | |
| RQ 3: What do we know about the effectiveness of existing research co-design processes? | |
|  | |
| Recommendations | |
| Made several recommendations for how to co-design with intellectually disabled populations, see page 280. | |

# Drahota et al., 2016: Community-Academic Partnerships: A Systematic Review of the State of the Literature and Recommendations for Future Research.

| Author(s) | Drahota, Amy; Meza, Rosemary D; Brikho, Brigitte; Naaf, Meghan; Estabillo, Jasper A; Gomez, Emily D; Vejnoska, Sarah F; Dufek, Sarah; Stahmer, Aubyn C; Aarons, Gregory A |
| --- | --- |
| Year | 2016 |
| Title | Community-Academic Partnerships: A Systematic Review of the State of the Literature and Recommendations for Future Research. |
| Source | The Milbank quarterly |
| DOI | 10.1111/1468-0009.12184 |
| AMSTAR (/11) | 6 |
| RQ 1: What research co-design processes exist in health settings? | |
| community-academic partnerships  CBPR | |
| RQ 2: What do these research co-design processes involve? | |
| Outlined several types of data collected during codesign including interviews, observations, surveys, meeting minutes/notes/agendas; field notes; focus groups and discussions. | |
| RQ 3: What do we know about the effectiveness of existing research co-design processes? | |
|  | |
| Recommendations | |
| The study provides several recommendations:  Facilitators to pursue:  Trust between partners  Respect between partners  Shared vision, goals and mission  Good relationship between partners  Effective and/or frequent communication  Well-structured meetings  Clearly differentiated roles and responsibilities  Good quality of leadership  Effective conflict resolution  Good selection of partners  Positive community impact  Mutual benefit for all partners  Hindering factors to avoid:  Excessive time commitment  Excessive funding pressures or control struggles  Unclear roles and/or functions of partners  Poor communication among partners  Inconsistent partner participation or membership  High burden of activities/tasks  Lack of shared vision, goals, and/or mission  Differing expectations of partners  Mistrust among partners  Lack of common language or shared terms among partners  Lack of common language or shared terms among partners  Bad relationship  See p. 184-186 for more detail. | |

# Cukor et al., 2016: Patient and Other Stakeholder Engagement in Patient-Centered Outcomes Research Institute Funded Studies of Patients with Kidney Diseases.

| Author(s) | Cukor, Daniel; Cohen, Lewis M; Cope, Elizabeth L; Ghahramani, Nasrollah; Hedayati, S Susan; Hynes, Denise M; Shah, Vallabh O; Tentori, Francesca; Unruh, Mark; Bobelu, Jeanette; Cohen, Scott; Dember, Laura M; Faber, Thomas; Fischer, Michael J; Gallardo, Rani; Germain, Michael J; Ghahate, Donica; Grote, Nancy; Hartwell, Lori; Heagerty, Patrick; Kimmel, Paul L; Kutner, Nancy; Lawson, Susan; Marr, Lisa; Nelson, Robert G; Porter, Anna C; Sandy, Phillip; Struminger, Bruce B; Subramanian, Lalita; Weisbord, Steve; Young, Bessie; Mehrotra, Rajnish |
| --- | --- |
| Year | 2016 |
| Title | Patient and Other Stakeholder Engagement in Patient-Centered Outcomes Research Institute Funded Studies of Patients with Kidney Diseases. |
| Source | Clinical journal of the American Society of Nephrology |
| DOI | 10.2215/CJN.09780915 |
| AMSTAR (/11) | NONE |
| RQ 1: What research co-design processes exist in health settings? | |
| community-based participatory research  Patient-Centered Research | |
| RQ 2: What do these research co-design processes involve? | |
| Table 2 provides detailed information on process for selecting stakeholders, types and frequency of interactions, and specific roles and responsibilities | |
| RQ 3: What do we know about the effectiveness of existing research co-design processes? | |
| Reports that "many of the patient representatives had very positive reactions to their time spent collaborating on the research projects. The representatives listed benefits to the studies due to their contributions, but also listed many personal benefits." (p.1711)  Some challenges reported included:  Finding time  Orientation/training needs  Transparency needs - knowing when and where inputs were used | |
| Recommendations | |
| They recommend "(1) defining the roles and process for the incorporation of input; (2) identifying the particular patients and stakeholders to build a foundation of key stakeholders who appropriately represent the target treatment population and its caregivers; (3) engaging patients and stakeholders so they appreciate the value of their own participation and have personal investment in the research process; and (4) overcoming barriers and challenges that arise and threaten the productivity of the collaboration." (p. 1711) | |

# Yoshida et al., 2016: Setting health research priorities using the CHNRI method: III. Involving stakeholders.

| Author(s) | Yoshida, Sachiyo; Wazny, Kerri; Cousens, Simon; Chan, Kit Yee |
| --- | --- |
| Year | 2016 |
| Title | Setting health research priorities using the CHNRI method: III. Involving stakeholders. |
| Source | Journal of global health |
| DOI | 10.7189/jogh.06.010303 |
| AMSTAR (/11) | NONE |
| RQ 1: What research co-design processes exist in health settings? | |
| The CHNRI method | |
| RQ 2: What do these research co-design processes involve? | |
| Discussed the use of the CHRNI prioritisation method (described here http://journals.plos.org/plosmedicine/article/file?type=supplementary&id=info:doi/10.1371/journal.pmed.1000389.s001 and here (https://www.researchgate.net/figure/CHNRI-methodology-process_fig1_308789383) | |
| RQ 3: What do we know about the effectiveness of existing research co-design processes? | |
|  | |
| Recommendations | |
|  | |

# Puts et al., 2017: Patient engagement in research with older adults with cancer.

| Author(s) | Puts, Martine T E; Sattar, Schroder; Ghodraty-Jabloo, Vida; Hsu, Tina; Fitch, Marg; Szumacher, Ewa; Ayala, Ana Patricia; Alibhai, Shabbir M H |
| --- | --- |
| Year | 2017 |
| Title | Patient engagement in research with older adults with cancer. |
| Source | Journal of geriatric oncology |
| DOI | 10.1016/j.jgo.2017.05.002 |
| AMSTAR (/11) | NONE |
| RQ 1: What research co-design processes exist in health settings? | |
| Patient and public involvement | |
| RQ 2: What do these research co-design processes involve? | |
|  | |
| RQ 3: What do we know about the effectiveness of existing research co-design processes? | |
| Impact on Patients  Positive: "feeling listened to and empowered, feeling valued, feeling part of a team, having improved access to information, being able to engage with researchers (which helped the patients understand research better and develop a more positive attitude towards research), and gaining a number of skills such as public speaking, group working and interviewing" (p.392)  Negative: "frustration due to feeling not valued or listened to, feeling marginalized, feeling not being taken seriously, [...] apprehension about engaging in something different, increased emotional burden due to having to recall their own experiences and listening to those of other patients" (p.392)  Impact on Researchers  Positive: "gaining new insights into the research issues and a greater understanding of patients' needs[...] greater diversity and sometimes even less workload for researchers, whose role changed from researcher to advisor."(p.392)  Negative "needing more time to engage patients, having to work on patient relationships, and needing more funds to implement patient engagement,conflicts when patients ideas did not match researchers visions, particularly to research practices. "Sensory and communication difficulties, the fluctuating health state of patient participants, cognitive impairment, dominance of some patients in meetings and low energy of patients to participate can be challenging for researchers [...]. Further challenges included: lack of predefined roles and expectations, difficulty sharing power, and dealing with patients who have with their own agenda." (p.392) | |
| Recommendations | |
| The paper provides many recommendations for engaging older patients with cancer in research (p. 394). Many of these may be generally applicable.  Conceptual phase  • The team should discuss what type of older adults with cancer should be on the team and what role(s) they would have. The  team should acquire the necessary resources to facilitate the engagement of older adults with cancer.  • Consider if one older adult or more should be recruited so that older adults on the team have an opportunity to share thoughts  and responsibilities with their peers. Older adults may not always be able to participate due to other commitments. Furthermore  if the research team is large it may be intimidating to be the only older adult on the team.  • Discuss how the team will measure the “impact” of engagement during the study to evaluate its effectiveness on study  outcomes and for the older adults involved so that future study design can benefit.  • Consider using reporting guidelines [...]] to document the engagement process and impact.  • Plan sufficient time to have valuable engagement.  Recruitment of older adults with cancer to be on the research team  • Recruitment of older adults should include efforts to reach the “hard-to-reach” (e.g. those with sensory impairments, mobility  impairments, and needing transportation assistance). The team should consider the logistics around the inclusion of older adults (location, time of day, use of technology required, etc.)  • Clearly explain to older adults what their role on the team will be (e.g. a collaborator who provides occasional input on the study progress or a research team member who would be involved in collecting data etc.) so that they know what is expected in terms of time commitments. At a minimum, their costs associated with participation should be reimbursed.  Team building  • Provide training for older adults. The training should explain what “research” is. Train researchers to work with older adults. Plan time for team building.  • Develop a communication plan for the entire team with a strategy in place on how to deal with disagreements and conflicts; consider including a neutral facilitator.  • Discuss with older adults with cancer how they would like to be informed about meeting materials: do they prefer the materials in advance or not, by email/post, do they want the scientific articles or not? Do they want to meet with researchers before meetings? How will they participate during the meetings (to avoid putting patients on the spot but to make sure they can bring their patient voice)? How will the team update the older adults during absences of team meetings to keep them engaged?  Design of the study  • The study research questions and goals should be set together by patients and researchers.  • Provide glossaries of terms and avoid jargon  During the study  • Depending on the role of the older adult(s), regular team meetings to evaluate progress towards study goals are needed  • Collect data to measure impact of the engagement of older adults on study outcomes  The researchers also recommend using the "The Guideline for Reporting Involvement of Patients and Public (GRIPP) ot Public Involvement Impact Assessment Framework (PiiAF) for planning, evaluating and reporting research co-design. | |

# Manafo et al., 2018: Patient engagement in Canada: a scoping review of the 'how' and 'what' of patient engagement in health research.

| Author(s) | Manafo, Elizabeth; Petermann, Lisa; Mason-Lai, Ping; Vandall-Walker, Virginia |
| --- | --- |
| Year | 2018 |
| Title | Patient engagement in Canada: a scoping review of the 'how' and 'what' of patient engagement in health research. |
| Source | Health research policy and systems |
| DOI | 10.1186/s12961-018-0282-4 |
| AMSTAR (/11) | 4 |
| RQ 1: What research co-design processes exist in health settings? | |
| Patient engagement | |
| RQ 2: What do these research co-design processes involve? | |
|  | |
| RQ 3: What do we know about the effectiveness of existing research co-design processes? | |
| Finds that there is a general lack of, strong, rigorous and generalisable studies to support claim that research co-design has positive impact on health outcomes and reform, stating that ""the content and quality of the articles providing this evidence was highly variable. Much of the purported benefits of patient engagement for impacting broader health outcomes and healthcare reform do not rely on experimental studies that have been assessed or formally evaluated by any formal measures of evaluation [...]. Several studies had limited or poorly described methods of assessment and evaluation [...]. Further, the studies that did evaluate impact were limited to qualitative analyses of small samples, which limits the generalisability of the findings [...]. In reference to SPOR’s identified desired outcomes, the literature most often revealed near or intermediate outcomes related to ‘being an active and informed partner in healthcare’, yet otherwise revealed limited impact on near, intermediate or long-term outcomes [...]. Often, the emphasis is on impacting the effectiveness and efficiency of the research process itself, with little to no translation across actual implementation and evaluation of research findings." (p. 7)  Also outlined a range of first personal reports of patient and researcher outcomes from engaging patients in health research:  Patients  "Patient developed own voice and agenda; patient was more prepared for broader collaboration with other stakeholder groups  Patients felt empowered, valued, and gained confidence and life skills  Researcher and patient developed improved trust  Improvement in information on all aspects of disease and treatment, involving patients in decision-making, organisation of care and the burden of neuropathy; setting  Improvement in quality of care in context of research priority setting" (p. 7)  Researchers  "Increased enrolment in studies and decreased attrition; improved data collection tools; improved dissemination of study findings and mobilisation of findings  Greater understanding and insight into research area; rapport with community built  Better alignment of research objectives through priority-setting activities  Improved research effectiveness  Improved opportunity to appraise and evaluate engagement opportunities in research" (p. 7) | |
| Recommendations | |
| Outlines successful engagement approaches for patient engagement in health research (p. 6)  1. Engage patients as early as possible and continue engagement throughout  2. Clearly define patient engagement plan; be clear on roles, duties and expectations between patients and researchers  3. Provide orientation and education about research and patient engagement  4. Provide ongoing support, encouragement and recognition for patient contributions  5. Facilitate mutual respect and valuing of patients’ expertise based on knowledge gained through experiences  6. Ensure a trusting and positive environment by providing structural support  7. Include a plan for evaluation of engagement  Also suggests shared characteristics of successful patient engagement in health research  • Clear purpose, role and structure for engaging patients  • Initiate and maintain partnerships between researchers and stakeholders  • Take the time required to foster relationship-building as the most critical component in establishing trust  • Clear leadership from principal investigator and/or wider culture of involvement  • Promote the need for facilitation of cross-communication among all groups  • Capture and optimise patient perspectives across all phases of research  • Ensure meaningful patient influence on research by validating the need for respect and support for patients  • Ensure adequate training for researchers and patients  • Share and promote research learnings, including evaluation efforts | |

# Schilling & Gerhardus, 2017: Methods for Involving Older People in Health Research—A Review of the Literature

| Author(s) | Schilling, Imke; Gerhardus, Ansgar |
| --- | --- |
| Year | 2017 |
| Title | Methods for Involving Older People in Health Research—A Review of the Literature |
| Source | International journal of environmental research and public health |
| DOI | 10.3390/ijerph14121476 |
| AMSTAR (/11) | 5 |
| RQ 1: What research co-design processes exist in health settings? | |
| patient and public involvement | |
| RQ 2: What do these research co-design processes involve? | |
| Reference groups, co-production, workshops, residencies, focus groups, surveys and postal feedback, sharing control, agenda-setting and partnering with researchers, feedback on understandability and appropriateness of documents, discussing and testing devices, identifying unanswered research questions,  See Tables 2 & 3 for more information. | |
| RQ 3: What do we know about the effectiveness of existing research co-design processes? | |
|  | |
| Recommendations | |
| Provided recommendations for involving older people that may apply to general research codesign.  Diversity  Involve a range of of diverse people but avoid tokenism  Use of separate PPI activities for people with different conditions and experiences  Communication  Use different strategies to enhance communication, such as ensuring accessibility of information, adapting information to the group [26,27], securing knowledge through refreshments and summaries [31], use of meaningful and non-suggestive task [30], setting a pace that is appropriate for all participants [27] and the use of visualizations [24,27,30,31].  Location  Choose a good location for conversation. Ideally accessible and familiar to participants.Consider need for transport.  a relaxed environment where involvement activities can take place unprejudiced [30] or sharing a neutral space with PPI participants for a few days spending both free and working time together were chosen [24]  Build Relationships  Manage hierarchies between researchers and participants can also contribute towards this effect.  Develop a personal relationship with participants  Allow enough time for both discussions and breaks  Emphasized flexibility as being very important  Tailor processes to the needs of the individuals  Invest time to get to know participants and their interests well, and also their strengths and weaknesses  Consider spending time with patients outside of data collection to build rappor  3.2.5 ?  Continuity  Expect that participants may come and go and have plan to maintain continuity of patient involvement  Use flexible PPI models (e.g., flexible attendance in meetings [26] or the use of different PPI methods interchangeably [28]) allowed the adjustment of involvement activities to the individual abilities of the participants and to alterations in the research process.  Support  Ensure that resources are put aside to provide support for Participants  Provide training for participants and a glossary of research terms  Use guides and prompts to direct co-researchers as needed  Flexible schedule for activities  Train moderation skills - the requirements can exceed normal levels of ability | |

# Bailey et al., 2015: Involving disabled children and young people as partners in research: a systematic review.

| Author(s) | Bailey, S; Boddy, K; Briscoe, S; Morris, C |
| --- | --- |
| Year | 2015 |
| Title | Involving disabled children and young people as partners in research: a systematic review. |
| Source | Child: care, health and development |
| DOI | 10.1111/cch.12197 |
| AMSTAR (/11) | 5 |
| RQ 1: What research co-design processes exist in health settings? | |
| Public and patient involvement (PPI) | |
| RQ 2: What do these research co-design processes involve? | |
| Discusses recruitment approaches for recruiting disabled children and young people (DCYP) that may apply to co-design more generally:  “Disabled children and young people had been accessed through schools, hospitals, in the community and online. Methods of advertising involvement opportunities included Internet forums, websites, newsletters and direct mailing. Partner organizations, youth workers and school staff were also used to reach young people. Some involvement opportunities required DCYP to volunteer directly to get involved, but one project required parents to get in touch on behalf of their child (Mogensen 2010). Only one project used a structured recruitment and selection process (VIPER 2013). Guidance recommends providing clear explanations of exactly what the DCYP are being asked to get involved in, using appropriate language in a variety of formats (Lightfoot & Sloper 2003; Street & Herts 2005).” (p. 510)  Table 2 outlines some of the ways in which DCYP have engaged in research co-design:  -Reviewed interview guides for interviewing DCYP. Contributed to policy recommendations to be included in the final report. Presented findings to local authority committees  -DCYP involved in the design of recruitment materials, interview topic guides, interpretation of results, planning a workshop for young participants and staff at which draft guidelines would be reviewed and finalized  -DCYP worked with professionals on the expert panel. They communicated with the team by email and social media. They contributed to the criteria and statements in the guide  - DCYP were involved in deciding where interviews were going to take place, who would be present at the interview, the topics to be covered in the interview, communication methods during the interview, and how they wanted to give and receive feedback  - DCYP could get involved in web?based activities or comment on drafts at fun days. They commented on artwork, format, size and types of information in the documents  - Involved in survey and qualitative research. Activities included: contributing to design of research, designing topic guides and questions for interview, carrying out interviews and focus groups, designing consent forms, data coding and analysis, preparing reports  - Developed research design and instruments. Also involved in implementation, analysis and dissemination. Data were coded by two trained youth community researchers. Random checks of these data were carried out to ensure accuracy  - Used disposable cameras to capture important things in their lives, advised researchers on issues that affect them | |
| RQ 3: What do we know about the effectiveness of existing research co-design processes? | |
| Figure 2 summarises the positive and negative impacts identified:  Potential positive impacts of involvement on DCYP  Increased confidence  Increased self-esteem  Gaining new skills and experiences  Greater responsibility and independence  Opportunity to socialize with peers  Enhanced knowledge of and access to decision making  Being empowered  Positive changes in expectations of being involved  An opportunity to share frustrations and appreciations  Knowing that their views and opinions are respected and valued  Knowing that they can make a difference  Knowing that their efforts may help other young people  Improved appropriateness and quality of research  Potential positive impacts of involvement on research  Prioritization of research questions that are relevant and important to DCYP  More age appropriate and accessible recruitment and advertising materials  Study protocols and interventions that are more acceptable to other DCYP4  Greater credibility and interest in studies  Positive contribution to data collection, especially when DCYP interview others in their peer group  A unique perspective during data analysis, particularly on data collected from other young people  Successful contribution to interview transcript coding  Potential positive impacts of involvement on DCYP  Learning about other’s lives and risk-taking behaviours may cause distress  Increased awareness of differences between how they view themselves and how others view then  Perceptions of tokenism may result in disillusionment and reduced benefits  It may not be possible to maintain confidentiality in meetings  May initially feel intimidated by unfamiliar people and working environment  May lose confidence or interest if faced with challenges they feel they cannot face  Potential negative impacts of involvement on research  Greater resources, staff skills and training are required to facilitate involvement  Research process may take longer | |
| Recommendations | |
| Provides recommendations for involving DCYP that may apply to general research codesign:  Anticipate and meet the communication and access needs of DCYP  If needed, use aids, such as drawings, photographs, talking mats, cue cards, pictures and tape recordings.  Consider involving a trusted adult or familiar communicator to facilitate and support communication  Ensure researchers have sufficient time, resources, skills and training for the engagement process  Ensure that venues are fully accessible and meet participants needs  Have appropriate timings for meeting with regular breaks, providing refreshments and sufficient time/support  Use engaging activities likely to be enjoyable, interesting and appropriate to age and/or ability  Incorporate flexibility and choice in activities to ensure that they are inclusive of different abilities  Acknowledge and thank DCYP for their time with payment, vouchers, outings or social occasions  Secure funding for costs associated with providing support for DCYP before recruiting  Give DCYP’s a chance to participate in research while protecting from feeling overburdened  Beware that sensitive meeting content can cause distress  Watch out for confidentiality issues in project meetings as it may be difficult to protect  Consider providing ongoing support after meetings  Build confidence  What for participants lacking confidence, or feeling intimidated or uncomfortable in new environments  Manage expectations of what involvement can achieve  Disarm concerns about not being listened to, token involvement, or inability to contribute  Allow participants to control certain tasks, define roles and raise important issues.  Be transparent about viable possibilities for change and outcomes from inputs received  Give feedback on outcomes of involvement  Increase responsibilities and expectations incrementally, as confidence grows | |

# Camden et al., 2015: Engaging stakeholders in rehabilitation research: a scoping review of strategies used in partnerships and evaluation of impacts.

| Author(s) | Camden, Chantal; Shikako-Thomas, Keiko; Nguyen, Tram; Graham, Emma; Thomas, Aliki; Sprung, Jennifer; Morris, Christopher; Russell, Dianne J |
| --- | --- |
| Year | 2015 |
| Title | Engaging stakeholders in rehabilitation research: a scoping review of strategies used in partnerships and evaluation of impacts. |
| Source | Disability and rehabilitation |
| DOI | 10.3109/09638288.2014.963705 |
| AMSTAR (/11) | 3 |
| RQ 1: What research co-design processes exist in health settings? | |
| Stakeholder engagement | |
| RQ 2: What do these research co-design processes involve? | |
| Outlines two strategies to recruit stakeholders for volunteer or paid roles:  Targeted, where "researchers selected the organizations or the individuals to be included. Direct invitations were made to partner organizations to nominate members on working committees" (p.1396)  Open, where "researchers asked partner organizations to disseminate the invitation to their members (e.g. by mail with a return stamped envelope) or used the media [...] For some paid positions, researchers drafted a job description delineating stakeholders’ roles, with opportunities to renegotiate roles later in the process" (p.1396)  Discusses the creation and management of roles and committees:  "The creation of one or various committees with different roles (e.g. working, steering or advisory committees, expert panel) was a strategy commonly used [...]. Stakeholders included persons with disabilities, caregivers, clinicians, researchers and support/community groups. They were reported to be active and engaged throughout the process. Roles of committee members included: reviewing the proposal and the results [...], being champions of the research program, liaising with research sites and adapting the research accordingly [...]. In some instances, stakeholders were involved in the whole research process, from setting the research agenda and research questions to data collection and analysis, and dissemination [...]. In two papers, stakeholders were considered as co-researchers [...]. Some committees participated in activities such as writing job descriptions, doing interviews and hiring personal, managing funds and organizing social events [...].  Committee activities included face-to-face and teleconference meetings and group discussions [...]. Frequency and duration of meetings varied across studies but it was perceived to be important to be able to keep stakeholders motivated and engaged. Buettgen et al. [...] reported having used face-to-face meetings when decisions needed to be taken, and phone meetings between face-to-face meetings to keep participants informed and engaged. In general, group discussions were held around themes such as service delivery issues, stakeholders’ roles and explanation of the research process in general (e.g. explaining the PAR steps to a co-researcher with a disability) [...].  Number of participants in those activities varied; some were open to all stakeholders interested while others were done with a smaller, selected group of participants. Written documents (e.g. presenting data to analyze or materials for dissemination) and flipcharts were reported as facilitating participation and were seen as useful to document the stakeholder engagement process [...]. Other important considerations for engaging stakeholders included scheduling meeting times and locations convenient for stakeholders (e.g. having meetings outside of the service-provider organization), engaging stakeholders in planning agendas, sharing the lead for activities among stakeholders, and outlining a plan for sustainability of group activities from the outset [...]" (p.1396)  Discusses processes involved in supporting stakeholders:  "It was perceived that stakeholders needed to be supported in order to understand research and to fulfill their role. Formal training and courses were used to build skills around different research components (e.g. research design, collecting data, facilitating meetings) [...] or to increase knowledge on different topics (e.g. client-centered services) [...]. The training occasionally integrated the use of videos to elicit discussions (e.g. about respectful relationships in the research context) [...]. Participants were sometimes paid to attend these training sessions [...]. Informal training was also reported to occur, mostly during committee meetings [...] or during data collection and analysis [...]. Using a specific framework to interpret data, debriefing field notes and hiring a research assistant as mentor were strategies used to increase research skills of the stakeholders. Key strategies found to integrate stakeholders in research are summarized in the Supplementary File 2." (p.1396)  Table 1 categorises the 19 studies examined based on the types of stakeholders involved and the research steps they engaged in.  Supplementary File 2 outlines the key strategies used for engagement:  Cognitive interviews  Focus/structured groups  Workshops  Regular teleconferences and/or in-person meetings  Sporadic meeting in specific steps of the research process at the stakeholder’s locations (e.g  school, community center)  Brainstorming sessions about outcomes and possible applications  Service users developed and conducted semi structured interviews  Stakeholders helped to design project's web page-members and provided feedback  Stakeholders paid as co-researchers  Stakeholders participating in steering/advisory committee  Training stakeholders to engage in research: formal and informal training  Use of different media and materials to ascertain engagement and understanding: lay summaries, flipchart, videos, drafts of project at different stages | |
| RQ 3: What do we know about the effectiveness of existing research co-design processes? | |
| Found that there was little work evaluating impacts.  "Of the articles retained, few used empirical data collection to identify factors influencing engagement or outcomes of engagement. Among these, no standardized measures were used, and the questionnaires, focus group guides and debriefing techniques used were not clearly described [...]. " (p. 1398)  Nonetheless suggested several impacts related to stakeholder engagement.  Creating partnerships and building value.  "A frequently mentioned impact was the creation of partnerships where each  participant learned to value different perspectives [...] Researchers can learn about the political system dynamics [...], the potential applications of research findings, and the lived experiences of stakeholders.[..] Service users and providers can gain insights on challenges related to service delivery, and immediate applications of research results [...]. Partnerships can also evolve into long-term collaborations where other projects can be generate[...] Families could also benefit from networking with others through engagement in research [...], and learn about ways of dealing with their members having a disability [...]. Some authors reported that partnerships could promote a model whereby theory, practice and research are interwoven to generate knowledge that will have important benefits for patient care" (p. 1397)  Making knowledge more easily applicable and facilitating the research process.  "Stakeholders’ engagement fostered identification of relevant questions, credibility of the knowledge produced and application of results adapted to contexts [...]. For example, services developed with stakeholders were more widely accepted and responsive to stakeholders’ needs [...] Specifically, engaging policy-makers helped secure funds for new services[...] while engaging individuals from patient support groups facilitated intervention delivery [14]. Engagement also helped adapting the study processes and materials, and facilitated the research process from recruitment to retention and dissemination of results [...]. Benefits were, however, questioned when stakeholders were consulted only at the end of the project" (p. 1397)  Empowerment.  "Stakeholders increased their confidence and skills, their awareness about specific needs and resources, their ability to advocate, to access information and social support [...]. Interactions between stakeholders also contributed to feelings of belonging to a group [...] " (p. 1397) | |
| Recommendations | |
| Makes several recommendations around specific aspects of engagement (p. 1396-1397):  Communication/culture  Expect different perspectives about stakeholders’ roles and expectations  Clarify and agree on realistic expectations at the beginning of the process  Find a balance between obtaining valid research results and meeting stakeholders’ goal (e.g. about their health conditions)  Have regular communication  Engage stakeholders in community based activities  Create spaces for voicing concerns  Assign clear roles  Have regular meetings  Create risk management strategies (i.e. what to do if problems arise)  Adapt scientific language and research materials to avoid jargon  Ensure everyone feels understood, comfortable and confident  Use flip charts and photos when appropriate to improve communication  Having stakeholders lead conversation can reduce cultural barriers and create a safe environment for the engagement of other participants  Watch for tension between stakeholder groups with different expectations.  Facilitate good group dynamics by planning (in grant proposals) enough time and opportunities to consult and understand group needs, include their feedback and adapt the materials  Power sharing  Consider the proportions of stakeholders involved.  Ensure that stakeholders, especially those in vulnerable populations, feel entitled to contribute at the same level as researchers Share control over the research process  Shifting ownership of the research process from researchers to stakeholders takes time  Incremental power sharing is recommended: stakeholders gradually take more decisions (e.g. setting meeting agendas or taking specific decisions about the research process)  Do not have fully predetermined roles and expectations for how stakeholders should participate - allow the stakeholders to determine how they would like to be engaged.  Dissemination should ensure stakeholders’ opinions are represented  Collaboration in identifying the research questions is crucial as it impacts the whole process, and influences the ownership over the project  Time, funding and resources.  Engaging stakeholders meaningfully required substantial time and financial commitments  Support stakeholders’ participation financially. Costs traveling expenses, training, support, administration, promotional activities, Knowledge Transfer, and special needs (e.g. adapting materials)  Plan, and budget, for sustainable stakeholder engagement: hire staff with time allocated to support stakeholder engagement, and maintain flexible timelines in the project  Other  Offer stakeholders the opportunity to be involved in dissemination and Knowledge Transfer. Identify their desired level of involvement and support it  Use the internet and social media to create a national database connecting stakeholders and researchers with similar interests  For recruitment for research co-design, they recommend "paying attention to the following criteria: (1) stakeholders’ characteristics (i.e. do they share key features of the group they represent), (2) stakeholders’ willingness to speak for the group they represent (as opposed to personal interests), (3) ability to communicate well, (4) achieving diversity in the group [...]. Identifying and engaging the right stakeholders was perceived as a challenge [...]; however partnering with organizations, providing salary and having a clear job description were factors perceived as facilitators for stakeholders’ identification and engagement" (p. 1396) | |

# Lee et al., 2017: Patient engagement in the design and execution of urologic oncology research.

| Author(s) | Lee, Daniel J; Avulova, Svetlana; Conwill, Ralph; Barocas, Daniel A |
| --- | --- |
| Year | 2017 |
| Title | Patient engagement in the design and execution of urologic oncology research. |
| Source | Urologic oncology |
| DOI | 10.1016/j.urolonc.2017.07.002 |
| AMSTAR (/11) | 3 |
| RQ 1: What research co-design processes exist in health settings? | |
| Patient engagement | |
| RQ 2: What do these research co-design processes involve? | |
| Outlines "possible actions to including patient coinvestigators":  -Identify potential need for patient coinvestigator  -Identify and apply for potential funding sources  -Delineate goals of patient coinvestigator and level of involvement  -Develop interface with patient survivor groups or patient liaisons  -Describe system for involvement: specific tasks, timeline, and outcomes"  Discusses several examples of specific cases of research co-design:  "The Comparative Effectiveness Analysis of Surgery and Radiation for localized prostate cancer (CEASAR) study was designed to compare the effectiveness of different management options for localized prostate cancer, focusing on PROs as the primary outcome measures. The study accrued over 3,000 men diagnosed in 2011 and 2012 and has followed them longitudinally. The study was funded first from the US Agency for Healthcare Research and Quality (AHRQ) and then from PCORI, with both funding sources requiring the perspective of numerous stakeholders in the design and execution of the study.  The research team initially attempted several techniques to engage patients, including creating a stakeholder panel, holding patient focus groups, and performing pilot testing and cognitive debriefing of study materials. These approaches were valuable initially in obtaining a broad array of viewpoints with respect to study endpoints and content of questionnaires. However, the process of holding large stakeholder meetings was somewhat unwieldy, and the study principal investigators recognized the need to identify a small number of patient members who were particularly engaged, whose input was most actionable, and who had the time to invest in weekly study meetings along with the other team members. In response to this, 2 individuals were invited to be “true” coinvestigators and to attend weekly meetings of the research team and become involved in the day-to-day management of the study. The involvement of patients in our research meetings had several noticeable effects on the research process. Initially, there was a need for “translation” from research jargon to lay language. This took a little bit of extra time and effort, but ultimately forced us to ensure that our study goals were explainable and meaningful to patients. Interaction with the patient members of the research team also forced the clinician researchers and biostatisticians to return frequently to the question of “how can this information be used by a patient?” As 1 example of the impact, we began to focus more on the clinical significance of differences between treatments (by identifying minimally important clinical differences on the scales we used) instead of statistical significance. Furthermore, the patient coinvestigators helped us make sure that the visual presentation of our results could be interpreted by patients, in order to maximize the utility of our publications to patients. The patient coinvestigators now play a critical role in the analysis, presentation, and dissemination of the CEASAR study data. In fact, it is their unique perspective that keeps the study “grounded” and ensures that we are generating useful information for patients (Table 2)."  "the Warwick Diabetes Care Research User Group enlisted a group of 26 patients with diabetes to join bimonthly meetings with their researchers [...]. This working group helped guide development of an electronic health portal to find relevant laboratory results and communicate with providers and other patients about their health status, and the development of clinical decision support tools to help patients make correct adjustments to their insulin requirements"  "Steffens et al. [...], who created a patient and family advisory council, consisting of patients who had undergone high-risk surgeries and relevant stakeholders. The patient and family advisory council identified the most common informational needs of elderly patients undergoing high-risk surgery, and then generated a question prompt list for patients to go through with their providers to help activate the patients in understanding more about the potential effect of their high-risk surgical procedures." | |
| RQ 3: What do we know about the effectiveness of existing research co-design processes? | |
|  | |
| Recommendations | |
|  | |

# Di Lorito et al., 2018: Co-research with adults with intellectual disability: A systematic review.

| Author(s) | Di Lorito, Claudio; Bosco, Alessandro; Birt, Linda; Hassiotis, Angela |
| --- | --- |
| Year | 2018 |
| Title | Co-research with adults with intellectual disability: A systematic review. |
| Source | Journal of applied research in intellectual disabilities |
| DOI | 10.1111/jar.12435 |
| AMSTAR (/11) | 5 |
| RQ 1: What research co-design processes exist in health settings? | |
| Co-Research  PPI | |
| RQ 2: What do these research co-design processes involve? | |
| Table 2 describes how co-research was conceptualised/executed in each of the 13 studies identified, these include: semi-structured interviews, observations and focus group sessions, qualitative interviews, video-recordings and personal diaries, focus groups sessions and semi structured questions, focus groups through questionnaires, field notes and observations, reflexive observations of authors, testing of inter-rate reliability response bias, testing inter-rater reliability, response bias consistency, test–re-test reliability and consistency of responses of co-researcher against academic researcher, reviewing literature, planning qualitative interviews, setting agendas, qualitative interviews, data analysis, and developing the questions of the qualitative interviews. | |
| RQ 3: What do we know about the effectiveness of existing research co-design processes? | |
| Outlines several positive impacts from research co-design  Benefits for co-researchers  Feeling more empowered and in control  Developing a more assertive attitude in expressing their views  Developing pride and accomplishment for being heard in a professional context  Becoming role models and advocates for their peers.  Develop skills during co-research that can be transferred used for future employment opportunities or in daily living  Extending their social and support network  Benefits for academic researchers  Changing expectations and assumptions on how to conduct research with adults with intellectual disability  Better understanding research roles  Understanding how each co-researcher has their own strengths and added value  Changing attitude towards co-researchers  Increasing awareness of benefits from patient involvement  Benefits for the research project  Co-researchers with relevant lived experience [...] benefit the research at all stages, for example, in creating materials for collecting data and disseminating results  Better data collection processes due to co-researchers increasing trust, putting peers/participants at ease and reformulating questions | |
| Recommendations | |
| Provides recommendations for research with intellectually disabled adults that may apply to general research codesign:  Provide research training.  Use accessible venues  Schedule meetings far in advance  Arrange travel and personal assistants if possible  Ensure the mental and physical well-being of all those involved  Provide adequate financial remuneration  Where needed use aids such as arrows and cards to aid co-researchers  Be aware of the the non-verbal cues of co-researchers as these may point to the co-researcher feeling overwhelmed or stressed  Use co-researchers to tailor the questions so they can be user-friendly, concrete, specific and relevant for participants  Figure 2 provides an example of good practice (see page 679)  Table 3 lists the facilitators identified in the 11 studies (see page 680 & 681) | |

# Fudge et al., 2007: Involving older people in health research.

| Author(s) | Fudge, N; Wolfe, C D A; McKevitt, C |
| --- | --- |
| Year | 2007 |
| Title | Involving older people in health research. |
| Source | Age and ageing |
| DOI | 10.1093/ageing/afm029 |
| AMSTAR (/11) | 3 |
| RQ 1: What research co-design processes exist in health settings? | |
| User involvement  Involving patients in research | |
| RQ 2: What do these research co-design processes involve? | |
| Table 1 gives information about specific roles and activities across 11 of studies included:  The paper also discusses rovides examples of process such as:  -Designing research studies, for example, "understanding concepts such as ‘quality of life’ and ‘quality of care’ from the perspective of the older person to develop appropriate research tools [...]; determining the acceptability of a randomised control trial for acute stroke treatment [...]; or consultation as part of the process of seeking approval for research projects by indigenous community groups [...]"  -Involvement in an advisory group. | |
| RQ 3: What do we know about the effectiveness of existing research co-design processes? | |
| Notes that "very few studies undertook evaluation of involving older people in research" and that "very little is known about how involvement changes research process, outcomes and quality"  The found that formal evaluation within their sample focused "primarily on the process of involving older people, using questionnaires or focus group discussions to identify ‘top tips’ for involving older people in research [...] or to determine the levels of satisfaction of taking part in such a process [...]"  The studies that conducted formal evaluation of research co-design generally found positive effects on participants, such as " research encouraged participants to become active in their community on issues of relevance to them" (p. 496). The studies that did not conduct formal evaluations found that "consultation with older people in the design stages resulted in a number of positive outcomes: one study was accepted by an ethics committee [...] and another by an indigenous community board [...]. Consultation also led to amore salient study design [...], resulted in discussion of issues researchers would not have anticipated [...], achieved better recruitment rates [...] and facilitated the consent process [...]. Authors of studies that did not undertake a formal evaluation also suggested that participants’ involvement led to: increased knowledge, awareness and confidence; meeting other people in similar situations; and the therapeutic value of being ‘listened to’ (p. 497) | |
| Recommendations | |
| Provides recommendations for involving older people that may apply to general research codesign.  Recruitment  Explore if older people can recruit others in social networks  Emphasise the benefits of involvement: "knowledge, sociable aspect, lunch, enjoyment, self-healing/therapeutic value, increasing confidence" (p. 498)  Expect that certain ethnic groups may feel ‘over-researched’ and have a negative perception of research, and therefore be reluctant to participate  Sustaining involvement  Ensure that you have suitable venues - not all participants will be conformation in religious venues  Make sure that adequate and culturally appropriate food is provided  Try to avoid excluding people with hearing problems  Expect and prepare for issues with power imbalances—tension, conflicts between users and researchers  Disarm service users concern that they cannot make a difference and that nothing will change despite the research occurring  If researchers giving something back to community they are working in, such as providing useful information, then the community is more likely to reciprocate and help with their research  Research activities  Build confidence and familiarity with research activities  Prepare for issues with Ill health, multiple medical conditions, hospital appointments, physical frailty and death  Prepare for issues relating to language barriers and jargon  Expect research users and researchers to have differing views of what constitutes a good study and what the purpose of research is  Expect research users and researchers to differ in their knowledge, experience, and priorities for the study outcomes  Expect that older people will have other commitments rather than assume their time is widely available  Prepare for user involvement to significantly alter the research timetable  Expect researchers to underestimate participants desire to be more actively involved  Expect researchers to underestimate participants capacity to be active co-researchers  Expect researchers to underestimate funding and time required for user involvement  People welcome the chance to discuss medical and health issues  Use focus groups and dramatisation to stimulate discussions  Focus groups allow discussion of unanticipated issues which can be incorporated into research design  Allow users rather than ‘experts’ to define research problems as this can encourage ownership and participation  Have a flexible agenda—allow issues outside of the research agenda to be discussed  Take time to build up partnerships and trust  The research team should commit to mediate cross-cultural and power imbalances  Provide training, information, orientation and a welcome package for participants | |

# Boote et al., 2010: Public involvement at the design stage of primary health research: a narrative review of case examples.

| Author(s) | Boote, Jonathan; Baird, Wendy; Beecroft, Claire |
| --- | --- |
| Year | 2010 |
| Title | Public involvement at the design stage of primary health research: a narrative review of case examples. |
| Source | Health policy |
| DOI | 10.1016/j.healthpol.2009.11.007 |
| AMSTAR (/11) | NONE |
| RQ 1: What research co-design processes exist in health settings? | |
| Public involvement | |
| RQ 2: What do these research co-design processes involve? | |
| "Group meetings were the most common method of public involvement. Contributions that members of the public made to research design were: review of consent procedures and patient information sheets; outcome suggestions; review of acceptability of data collection procedures; and recommendations on the timing of potential participants into the study and the timing of follow-up."  "Each paper reported that group meetings with patients and/or members of the public were held to discuss research design issues. These were described as either focus groups or consultation meetings. Participants of these meetings were either specially convened by the researcher [30,33] or comprised members of existing support groups [...]. Focus groups or consultation meetings with existing support groups have the advantage of allowing researchers preparing bids for funding, where timescales are often very short, the opportunity to consult with interested patients, service users and members of the public in a relatively short period of time. As Marsden and Bradburn state, “focus group methodology was selected as it is less timeconsuming than individual interviews but yields similar information” [...]. Other methods of public involvement in research design cited include in-depth interviews, home observations and taste trials [...]; telephone conversations and questionnaires [...]; and a stakeholder event to reach consensus [...]."  Key contributions from members of the public: "review of patient information sheets and consent procedures; suggestion of outcome measures; review of acceptability of data collection procedures; and recommendations on the timing of potential participants into the study and timing of follow-up."  Table 2 describes the codesign processes used in the 7 studies included. | |
| RQ 3: What do we know about the effectiveness of existing research co-design processes? | |
|  | |
| Recommendations | |
| Provided several recommendations:  Expect and disarm tensions between different stakeholder groups  Prepare for issues with members of the public failing to understand health research methods  Expect that involvement will require extra time and cost  Expect and prepare for issues in ensure proper representativeness  Expect and minimise issues with the use of research language and jargon  Ensure cultural sensitivity when collecting data  Provide training and make sure to explain health research methods to co-researchers  Use independent facilitation if many different types of stakeholders are involved  Having funding to pay participants for their involvement in research co-design | |

# Oliver et al., 2004: Involving consumers in research and development agenda setting for the NHS: developing an evidence-based approach.

| Author(s) | Oliver, S; Clarke-Jones, L; Rees, R; Milne, R; Buchanan, P; Gabbay, J; Gyte, G; Oakley, A; Stein, K |
| --- | --- |
| Year | 2004 |
| Title | Involving consumers in research and development agenda setting for the NHS: developing an evidence-based approach. |
| Source | Health technology assessment |
| DOI | 10.1017/S026646230400073X |
| AMSTAR (/11) | 4 |
| RQ 1: What research co-design processes exist in health settings? | |
| Consumer involvement | |
| RQ 2: What do these research co-design processes involve? | |
| Report discusses many different types of research co-design process, including committee membership, team working, written and face-to-face consultations, surveys, large scale meetings and  consumer activism.  Methods discussed include "written consultations, face-to-face consultations, committee membership and indirect methods such as scanning consumer literature, and questions to helplines and Members of Parliament."  Tables 1-8 and Appendix 2 summarises the processes involved for co-design within 135 studies.  Argue that co-design can be classified into four levels of research engagement and four levels of consumer engagement (see Figure 1). Each of these different combinations of enagement is associated with different processes for data collection and involvement. | |
| RQ 3: What do we know about the effectiveness of existing research co-design processes? | |
| They outline outcomes and processes that may be assessed in attempts to evaluate consumer involvement in setting research agendas in Box 3.  Benefits for intermediate measures  More informed discussion between consumers and professionals  Greater understanding by consumers of technical issues.  Improved sensitivity to consumer concerns in planning research.  Reduced barriers to broad participation.  Improved public profile of research.  Increased credibility of consumers.  Increased enthusiasm amongst consumers and professionals for working together.  Consumer and manager descriptions of success  in involving consumers in R&D  Benefits for final measures  Research incorporating consumers’ ideas and addressing consumers’ concerns and needs.  Greater uptake of research findings.  Improved care.  Improved health.  Cost/harms for intermediate measures  Increased workload.  Decreased enthusiasm amongst consumers and professionals for working together.  Greater confusion about the purpose of research.  Damaged public profile of research.  Reduced credibility of consumers.  Lack of reporting of consumer involvement.  Cost/harms for final measures  Delayed research.  More expensive research.  Disappointment in limitations of research.  Reduced uptake of research findings.  Argue that "what we know about the advantages and disadvantages of methods for involving consumers in agenda setting rests on weak shortterm evidence and almost entirely speculative long-term evidence" (p. 102) | |
| Recommendations | |
| Most of the recommendations focused on research. However, the following facilitators for involving consumers in agenda setting were also outlined:  Support from leadership  Broad end-user involvement  Building on existing working relationships  Encouragement and support  Good communication  Providing event related information well in advance  Face-to-face and telephone contact rather than letters and emails  Use of plain english rather than jargon  Training and support for participants - ideally complementing practical research experience  Planning and budgeting for sufficient resources in advance  Building on experience and developing ongoing relationships | |

# Boote et al., 2011: Public involvement in the systematic review process in health and social care: a narrative review of case examples.

| Author(s) | Boote, Jonathan; Baird, Wendy; Sutton, Anthea |
| --- | --- |
| Year | 2011 |
| Title | Public involvement in the systematic review process in health and social care: a narrative review of case examples. |
| Source | Health policy |
| DOI | 10.1016/j.healthpol.2011.05.002 |
| AMSTAR (/11) | NONE |
| RQ 1: What research co-design processes exist in health settings? | |
| Public involvement | |
| RQ 2: What do these research co-design processes involve? | |
| Identified five contributions that the public can make to the systematic review process: "(1) refining the scope of the review; (2) suggesting and locating relevant literature; (3) appraising the literature; (4) interpreting the findings; (5) writing up the review"  Also suggested that there are three levels of public involvement in research: 'consultation', 'collaboration', and 'user led'.  Table 2 outlines specific actors and co-design processes within seven research projects. | |
| RQ 3: What do we know about the effectiveness of existing research co-design processes? | |
|  | |
| Recommendations | |
| Makes several recommendations for co-design in the systematic review process.  Expect to underestimate the time required - ensure that there is sufficient time for all desired research activities and anticipate delays due to logistic and coordination challenges.  Expect to underestimate the funding required - ensure that there is sufficient finding for all desired research activities and anticipate costs for funding involvement, coordination and logistics. Pay participants for involvement and cover out of pocket expenses. Discuss and finalise payment early on and implement in budget. Expect that payment for involvement may affect participant benefits and will take time to set up  Expect and prepare for issues with continuity as not all initial participants will be able to be involved throughout the entire process  Expect and prepare for issues with group dynamics. Trust will take time to develop. Different groups may try to dominate others.  Anticipate issues with Research Ethics Committees. These may treat involved patients as research participants rather than advisors and seek informed consent.  Anticipate and prepare for issues with selection of participants. Participants may consider themselves unworthy to represent organisations or patient groups.  A member of the research team should take lead responsibility for public involvement. This time should be adequately budgeted  Provide sufficient training and information to the participants. This information might include, for example, a glossary of key terms, background information and briefing notes  Use structured methods of involvement to refine the population, interventions and outcomes of interest. These may include the nominal group technique, the Delphi method, and voting within review advisory group meetings.  Table 2 (p.110 - 111) describes recommendations made by the studies included. | |

# Oliver et al., 2008: A multidimensional conceptual framework for analysing public involvement in health services research.

| Author(s) | Oliver, Sandy R; Rees, Rebecca W; Clarke-Jones, Lorna; Milne, Ruairidh; Oakley, Ann R; Gabbay, John; Stein, Ken; Buchanan, Phyll; Gyte, Gill |
| --- | --- |
| Year | 2008 |
| Title | A multidimensional conceptual framework for analysing public involvement in health services research. |
| Source | Health expectations : an international journal of public participation in health care and health policy |
| DOI | 10.1111/j.1369-7625.2007.00476.x |
| AMSTAR (/11) | 4 |
| RQ 1: What research co-design processes exist in health settings? | |
| Public involvement | |
| RQ 2: What do these research co-design processes involve? | |
| See Oliver 2004 | |
| RQ 3: What do we know about the effectiveness of existing research co-design processes? | |
| See Oliver 2004 | |
| Recommendations | |
| See Oliver 2004 | |

# Boote et al., 2012: Involving the public in systematic reviews: a narrative review of organizational approaches and eight case examples

| Author(s) | Boote, J.; Baird, W.; Sutton, A. |
| --- | --- |
| Year | 2012 |
| Title | Involving the public in systematic reviews: a narrative review of organizational approaches and eight case examples |
| Source | Journal of comparative effectiveness research |
| DOI | 10.2217/cer.12.46 |
| AMSTAR (/11) | NONE |
| RQ 1: What research co-design processes exist in health settings? | |
|  | |
| RQ 2: What do these research co-design processes involve? | |
| Present similar claims to Boote, 2011  Table 2 outlines specific actors and co-design processes within eight research projects. | |
| RQ 3: What do we know about the effectiveness of existing research co-design processes? | |
|  | |
| Recommendations | |
| Outlines several recommendations for co-design in review, most of which mirror those discussed in Boote 2011. Two additional recommendations are:  Where necessary prepare the research team for how to interact with patients and how to discuss difficult subjects with them  Consider training researcher for public engagement through consultation with relevant experts or organisations | |

# Morley et al., 2016: A systematic scoping review of the evidence for consumer involvement in organisations undertaking systematic reviews: focus on Cochrane

| Author(s) | Morley, Richard F.; Norman, Gill; Golder, Su; Griffith, Polly |
| --- | --- |
| Year | 2016 |
| Title | A systematic scoping review of the evidence for consumer involvement in organisations undertaking systematic reviews: focus on Cochrane |
| Source | Research Involvement and Engagement |
| DOI | 10.1186/s40900-016-0049-4 |
| AMSTAR (/11) | 4 |
| RQ 1: What research co-design processes exist in health settings? | |
| Consumer involvement | |
| RQ 2: What do these research co-design processes involve? | |
| Table 2 gives information about research co-design processes identified. The include advisory group membership, editorial team membership, and the evaluation of the relevance of research questions. | |
| RQ 3: What do we know about the effectiveness of existing research co-design processes? | |
| Argued that three "general general impacts were identified across studies: increased relevance and timeliness; better dissemination including greater accessibility, and reduction in bias" (p. 13)  Noted that "the intangible nature of some of the benefits that were documented may explain some of the lack of certainty about impact which was documented in many of the studies" (p. 14) | |
| Recommendations | |
|  | |

# Brett et al., 2010: The PIRICOM Study: A systematic review of the conceptualisation, measurement, impact and outcomes of patients and public involvement in health and social care research

| Author(s) | Brett, Jo; Staniszewska, Sophie; Mockford, Carole; Seers, Kate; Herron-Marx, Sandra; Bayliss, Helen |
| --- | --- |
| Year | 2010 |
| Title | The PIRICOM Study: A systematic review of the conceptualisation, measurement, impact and outcomes of patients and public involvement in health and social care research |
| Source | University of Warwick |
| DOI |  |
| AMSTAR (/11) | 7 |
| RQ 1: What research co-design processes exist in health settings? | |
| PPI | |
| RQ 2: What do these research co-design processes involve? | |
| Provides a considerable amount of information into the processes involved in research co-design.  Table 5 summarises 13 studes which reported outcomes on reserach codesign, and discusses processes, roles and outcomes.  Appendix 3 summarises all 98 documents included in the report and oultines how co-design occured in each. | |
| RQ 3: What do we know about the effectiveness of existing research co-design processes? | |
| Provides a detailed evaluation of impacts of research co-design.  Summarises the overall Public and Patient Involvement evidence base as:  "The evidence base underpinning PPI in health and social care research is complex reflecting the wide diversity of the PPI landscape and activity [is] comprised of mainly qualitative or case study reflections of PPI, or crosssectional studies reporting individual or organisational views of PPI, with relatively little critical evaluation [and that] the impact and outcomes of PPI are [mainly represented] through narrative description, which is usually too brief to provide a full understanding of impact" (p. 11 & 12)  Also comments that "The evidence base appears to be relatively weak in relation to the quality and detail of impact reporting, and needs significant enhancement. However, this may reflect the timescale of the review which has included studies from the last 15 years and may reflect times when the current interest in evaluating impact was not present in the same way" (p. 12)  Argues that "a comprehensive theoretical model of PPI that can be empirically tested a blueprint for the development of instruments to capture or measure impact" (p. 12)  Suggests that despite limitations in the evidence base, the following PPI impacts were identified:  Impact on research and research process  "Examples of PPI impacts in relation to research and the research process have been found in the initial stages of research, such as developing research questions, identifying and prioritising topics, developing commissioning briefs. In undertaking research, there was evidence of impact on developing and commenting on research protocols, adapting and improving the sensitivity of research language in information and invitation letters and identifying poorly worded questions in questionnaires." (p. 12 & 13)  "There is evidence that PPI helps build important links with the community and can help with accessing participants, improving response rates, recruitment from seldom heard groups, development of greater empathy with research subjects and better informed consent based on a more informed participant. There is also evidence that PPI can help in the assessment and development of research instruments, improve the timing of interventions and ensure the instruments are more acceptable to the community. Users can also collect deeper and more insightful data based on their rapport with the participant. There is also evidence of impact on data analysis with users providing a wider perspective, different insights and identifying knowledge gaps for future research. PPI can also impact on dissemination and implementation due to the dedication of users, and in some cases through the development of a cohort of advocates who disseminate key findings." (p. 13)  Impact of PPI on users  "The beneficial impacts were divided into three main areas: personal benefits, impact on level of knowledge and impact on their level of skill. Personal benefits include feeling empowered, feeling listened to, feeling more positive, feeling more confident, and feeling a sense of fulfilment and satisfaction. Users felt mutual support from being part of a team and appreciated the social interaction with others. Users also felt they had given something back and had done something meaningful for the research community and felt they had made a difference. Users also reported improved levels of knowledge, more open attitudes to research and improved trust in research. Some users reported access to better information about their condition and enhanced capacity to manage their condition and solve related problems. Users also reported positive impacts in relation to gaining skills in research methodology and in gaining other skills such as confidence in speaking, and listening in groups. Some papers reported more negative impacts in terms of personal impacts, skills levels and knowledge levels. For example users reported feeling overburdened, not listened to, frustrated and marginalised" (p. 13)  Other areas of impact  "Most evidence of impact related to research and the research process and to users, with much less impact reported in relation to researchers, researcher participants, community, policy makers, journals and funders. The detail of these impacts is reported in the results section " (p. 14)  Impact of PPI on outcomes  "Studies reported beneficial outcomes (the results of PPI in a study) to the development of research agendas, aims and priorities. These include the following main areas of outcomes: new research, research questions or topic areas identified; new research proposals suggested or developed; new types of medication developed; cultural equivalence of research tools enhanced; context of care and impact on provision of services considered; research gaps identified and development of future research designs. (p. 14)  “Studies reported beneficial outcomes on a range of aspects of study design including applicability of informed consent, improved design of a trial, judging whether the climate was right for a study, deciding on appropriate endpoints, appropriate time for recruitment, interpretation of information for participants in a study and outcome measure selection.”(p. 14)  Economic analysis  "There was no evidence of economic analysis, reflecting the lack of appraisal of the impact of PPI more generally" (p. 14)  Pages 46 - 93 provide a detailed discussion of the positive and negative impacts of PPI on the research and research process, users, researchers, research participants, community, funders and policy makers.  Pages 120 & 121 outline PIRICOM Guidelines for reporting PPI Impact. | |
| Recommendations | |
| Table 2, pages 47-48, make 16 recommendations for involvement:  1. Budget appropriately for the service users involvement. This may include contributions to service users for their time, expenses, cost of training etc  2. Consider additional time needed for PPI activity in time scales for the study  3. Involve service users as early as possible in the research, preferably at the beginning of the study and maintain involvement throughout  4. Define roles of service users and researchers in the PPI activity  5. Provide service users adequate training on research skills required for their involvement in the study. Provide service users with the additional knowledge of the disease/condition that is necessary in order for them to contribute  6. Provide researcher with training on how to involve service users in research and encourage a positive attitude to PPI  7. Establish good relationships between service users and researchers over  time, and avoid recruiting service users in a hurry  8 Respect the skills, knowledge and experience that service users bring to a research study  9. Provide personal support and supervision of service users  10. Ensure good communication to manage conflict and avoid isolation  10. Involve service users in developing invitation letters, information sheets, consent forms, questionnaires, interview schedules – as service users will assist in developing this information in a patient-relevant way  11. Involve service users in decisions as to how participants are recruited  12. If sufficient training is provided, service users can assist in data collection  13. Service users can identify patient-important themes in the data  14. Detail in reports/publications how PPI was conducted  15. Produce a lay summary of the final report so it can be easily understood  by the target population  16. Develop service user advocacies for dissemination and implementation of research to assist in making the results more poignant and more relevant to the target population | |
