# Supplementary material for: Research co-design in health: a rapid overview of reviews
Source: Health Res Policy Syst. 2020 Feb 11;18:17. doi: 10.1186/s12961-020-0528-9 (PMC7014755; doi:10.1186/s12961-020-0528-9)
Supplement: Supplementary file 6 — Additional file 6: Quality assessment [file 12961_2020_528_MOESM6_ESM.docx]

Additional file 6: Quality assessment

Quality assessment was conducted using AMSTAR (2007, 2009)

- Shea BJ, Grimshaw JM, Wells GA, Boers M, Andersson N, Hamel C, Porter AC, Tugwell P, Moher D, Bouter LM. *Development of AMSTAR: a measurement tool to assess the methodological quality of systematic reviews*. BMC Med Res Methodol. 2007 Feb 15; 7:10. PMID: 17302989.
- Shea BJ, Hamel C, Wells GA, Bouter LM, Kristjansson E, Grimshaw J, Henry DA, Boers M. *AMSTAR is a reliable and valid measurement tool to assess the methodological quality of systematic reviews*. J Clin Epidemiol. 2009 Oct; 62(10):1013-20. PMID: 19230606

| **AMSTAR question** | **Record** | | | | | | |
| --- | --- | --- | --- | --- | --- | --- | --- |
|  | **Oliver 2004/2008** | **Domecq 2014** | **Haijes 2016** | **Drahota 2016** | **Manafo 2018** | **Schilling 2017** | **Bailey 2015** |
| 1. Was an 'a priori' design provided? | No | Yes | No | Yes | No | Yes | No |
| 2. Was there duplicate study selection and data extraction? | Can't answer | Can't answer | No | Yes | No | No | Yes |
| 3. Was a comprehensive literature search performed? | Yes | Yes | Yes | Yes | Yes | Yes | Yes |
| 4. Was the status of publication (i.e. grey literature) used as an inclusion criterion? | Yes | Yes | No | No | Yes | No | Yes |
| 5. Was a list of studies (included and excluded) provided? | No | No | No | No | No | No | No |
| 6. Were the characteristics of the included studies provided? | Yes | Yes | Yes | Yes | Yes | Yes | Yes |
| 7. Was the scientific quality of the included studies assessed and documented? | No | Yes | No | No | No | No | No |
| 8. Was the scientific quality of the included studies used appropriately in formulating conclusions? | No | Yes | No | No | No | No | No |
| 9. Were the methods used to combine the findings of studies appropriate? | Yes | Yes | Yes | Yes | Yes | Yes | Yes |
| 10. Was the likelihood of publication bias assessed? | No | No | No | No | No | No | No |
| 11. Was the conflict of interest included? | No | No | No | Yes | No | Yes | No |
| **Total** | 4 | 7 | 3 | 6 | 4 | 5 | 5 |

| **AMSTAR question** | **Record** | | | | | |
| --- | --- | --- | --- | --- | --- | --- |
|  | **Camden 2015** | **Lee 2017** | **DiLorito 2018** | **Fudge 2007** | **Morley 2016** | **Brett 2010/2014** |
| 1. Was an 'a priori' design provided? | No | No | No | No | No | No |
| 2. Was there duplicate study selection and data extraction? | Can't answer | Can't answer | No | No | No | No |
| 3. Was a comprehensive literature search performed? | Yes | Yes | Yes | Yes | Yes | Yes |
| 4. Was the status of publication (i.e. grey literature) used as an inclusion criterion? | No | No | No | No | No | Yes |
| 5. Was a list of studies (included and excluded) provided? | No | No | No | No | Yes | Yes |
| 6. Were the characteristics of the included studies provided? | Yes | Yes | Yes | Yes | Yes | Yes |
| 7. Was the scientific quality of the included studies assessed and documented? | No | No | Yes | No | No | Yes |
| 8. Was the scientific quality of the included studies used appropriately in formulating conclusions? | No | No | No | No | No | Yes |
| 9. Were the methods used to combine the findings of studies appropriate? | Yes | Yes | Yes | Yes | Yes | Yes |
| 10. Was the likelihood of publication bias assessed? | No | No | No | No | No | No |
| 11. Was the conflict of interest included? | No | No | Yes | No | No | No |
| **Total** | 3 | 3 | 5 | 3 | 4 | 7 |
